# Investigation of Inflammation and Tissue Patterning in the Gut Using a Spatially Explicit General-Purpose Model of Enteric Tissue (SEGMEnT)

**DOI:** 10.1371/journal.pcbi.1003507

**Published:** 2014-03-27

**Authors:** Chase Cockrell, Scott Christley, Gary An

**Affiliations:** Department of Surgery, University of Chicago, Chicago, Illinois, United States of America; Johns Hopkins University, United States of America

## Abstract

The mucosa of the intestinal tract represents a finely tuned system where tissue structure strongly influences, and is turn influenced by, its function as both an absorptive surface and a defensive barrier. Mucosal architecture and histology plays a key role in the diagnosis, characterization and pathophysiology of a host of gastrointestinal diseases. Inflammation is a significant factor in the pathogenesis in many gastrointestinal diseases, and is perhaps the most clinically significant control factor governing the maintenance of the mucosal architecture by morphogenic pathways. We propose that appropriate characterization of the role of inflammation as a controller of enteric mucosal tissue patterning requires understanding the underlying cellular and molecular dynamics that determine the epithelial crypt-villus architecture across a range of conditions from health to disease. Towards this end we have developed the Spatially Explicit General-purpose Model of Enteric Tissue (SEGMEnT) to dynamically represent existing knowledge of the behavior of enteric epithelial tissue as influenced by inflammation with the ability to generate a variety of pathophysiological processes within a common platform and from a common knowledge base. In addition to reproducing healthy ileal mucosal dynamics as well as a series of morphogen knock-out/inhibition experiments, SEGMEnT provides insight into a range of clinically relevant cellular-molecular mechanisms, such as a putative role for Phosphotase and tensin homolog/phosphoinositide 3-kinase (PTEN/PI3K) as a key point of crosstalk between inflammation and morphogenesis, the protective role of enterocyte sloughing in enteric ischemia-reperfusion and chronic low level inflammation as a driver for colonic metaplasia. These results suggest that SEGMEnT can serve as an integrating platform for the study of inflammation in gastrointestinal disease.

## Author Summary

Mucosal histology plays a key role in the diagnosis, characterization and propagation of a host of gastrointestinal diseases, and the development of computational models capable of producing spatial architecture comparable to histology will enhance the evaluation of hypotheses for those diseases. Inflammation is a significant factor in the pathogenesis of a series of gastrointestinal diseases, and affects the maintenance of the mucosal architecture by morphogenic pathways. We have developed the Spatially Explicit General-purpose Model of Enteric Tissue (SEGMEnT) to investigate the behavior of enteric epithelial tissue as influenced by inflammation. SEGMEnT integrates cellular and molecular pathways governing morphogenesis and inflammation to generate a variety of pathophysiological processes from a common platform and knowledge base. Beyond reproducing healthy and disease ileal mucosal dynamics, SEGMEnT provides insight into a range of clinically relevant cellular-molecular mechanisms, including a novel putative role for Phosphotase and tensin homolog/phosphoinositide 3-kinase (PTEN/PI3K) as a key point of crosstalk between enteric inflammation and morphogenesis, the protective role of enterocyte sloughing in enteric ischemia-reperfusion and the mechanism of chronic low level inflammation as a driver for colonic metaplasia. These results suggest that SEGMEnT can serve as an integrating platform for the study of inflammation in gastrointestinal disease.

## Introduction

**Figure 1:**
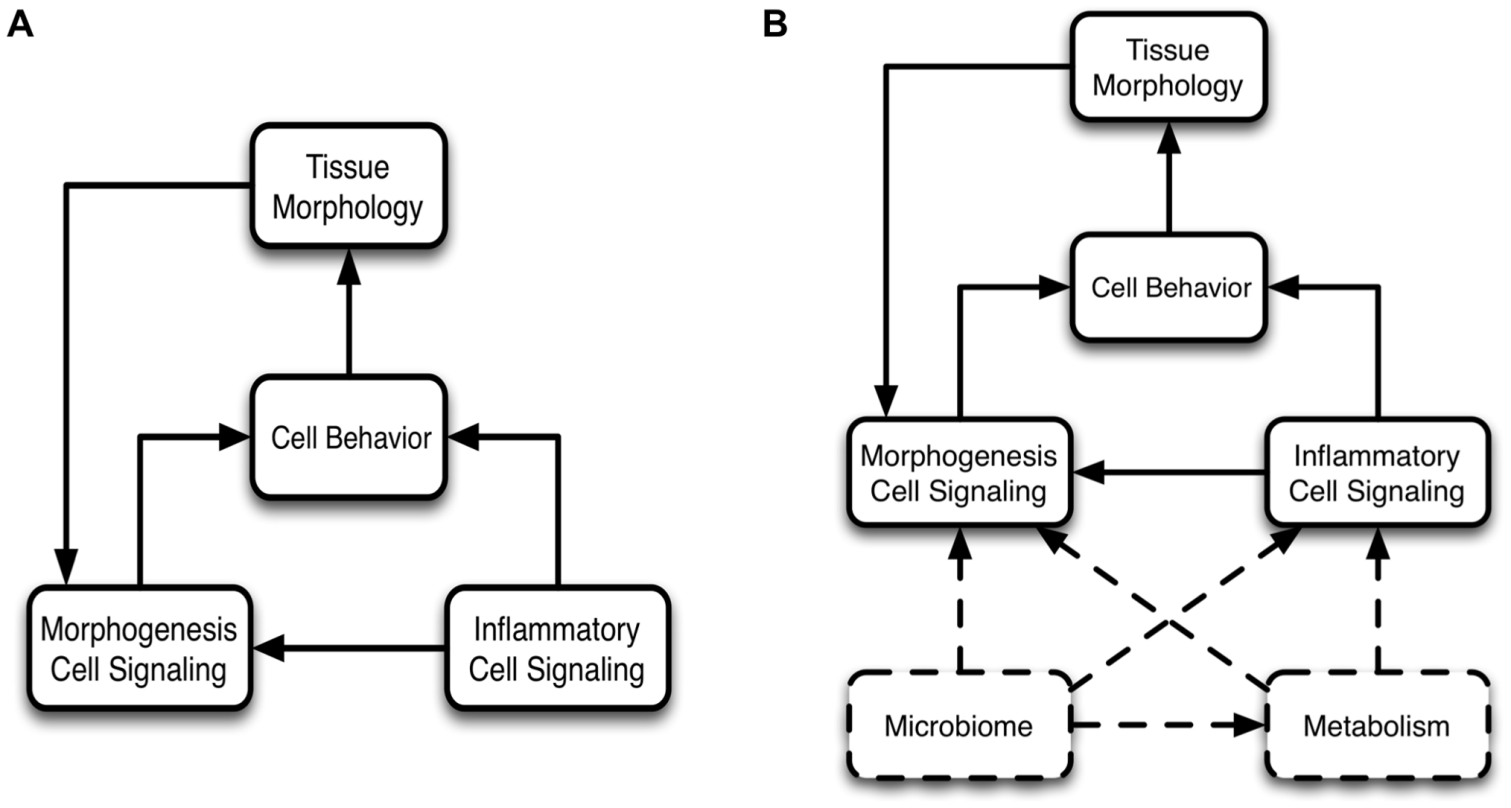
Modular control structure for SEGMEnT. A simple representation of SEGMEnT's modular control structure is shown in Panel A: morphogenesis cell signaling determines cell behavior, the aggregate of which defines the tissue morphology. An inflammatory module acts as a controller on the morphogenesis processes. Because of the structure/function relationship in gut epithelial tissue the tissue morphology further modulates the morphogenesis cell signaling. Panel B demonstrates future planned control modules to be added to SEGMEnT in order to expand its functional representation (boxes and connectors with dashed lines).

The gut epithelium faces unique challenges in striking a balance between its receptive role in the absorption of nutrients and fostering synergistic interactions with commensal microbes versus retaining sufficient defensive barrier function to prevent microbial invasion and heal tissue injury effectively within this complex environment. The clinical relevance of the structure-function relationship of the gut mucosa is readily evident in the fact that histological characterization of intestinal mucosal architecture is a mainstay in the diagnosis of intestinal disease [1]. We propose that a broad spectrum of intestinal disease can be unified by a view that the mucosal tissue architecture, as maintained by morphogenesis pathways, is subject to a series of control modules that effectively balance the complex interplay of multiple functional objectives when in a state of health, but can become disturbed to generate pathological conditions (Figure 1). Of these control modules, inflammatory pathways are among the most clinically significant, playing an important pathophysiological role in a host of intestinal diseases ranging from environmental enteropathy [2], necrotizing enterocolitis [3], inflammatory bowel disease [4], gut-derived sepsis [5] and cancer [6]. While the specific manifestation of gut inflammation may be different in each of these situations, the same general set of processes are involved across this spectrum of diseases, and are correspondingly associated with specific histological changes of the gut mucosa. Given the complexity of the processes and interactions present, dynamic computational modeling can be a useful tool for instantiating conceptual models (hypotheses) of the structure-function relationship in the gut and its role in the pathogenesis of gastrointestinal disease. Towards this end, we have developed an agent-based computational model to simulate the cellular and molecular interactions that maintain and modify the enteric mucosal architecture, the Spatially Explicit General-purpose Model of Enteric Tissue (SEGMEnT). SEGMEnT models the spatial dynamics of the crypt-villus tissue architecture as generated by the behavior of gut epithelial cells as they undergo replication, migration and differentiation, with the novel incorporation of the effect of inflammation on those morphogenic processes (Figure 1).

SEGMEnT provides a platform for instantiating conceptual models (hypotheses) representing baseline, healthy systems, which can then be used to explore, through differential perturbations, different disease trajectories [7]–[9]. Previous work in spatially-explicit modeling of the gut epithelium is broadly split into two categories: 1) models depicting de novo morphogenesis to examine the generative processes governing crypt-villus growth and patterning [10]–[13], or 2) models with a fixed crypt architecture that considers corresponding issues such as morphogen spatial distribution [14], [15], cell type distribution [16]–[18], and homeostasis [17], [18]. We adopt a distinct but complementary approach that combines aspects of both of these approaches, where we assume the existing tissue architecture has already been produced and the morphogenic pathways maintain the subsequent homeostatic state. However, we allow for a mutable morphologic topology, specifically via alterations in the crypt-villus configuration and tissue architectural features (e.g., the location of the crypt-villus junction, relative and absolute sizes of the crypt and villus). This capability provides metrics for comparing SEGMEnT's output with various histological phenotypes representing different states of health and disease in the gut. Furthermore, we incorporated inflammation as an additional controller of morphogenesis, thereby providing the means to represent many different pathophysiological processes and outcomes.

**Figure 2:**
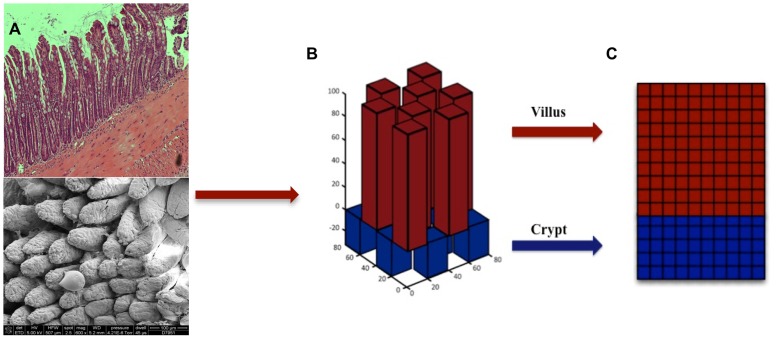
SEGMEnT topology. Panel A contains a histology cross section of ileal tissue (top) and scanning electron microscopy of the mucosal surface of ileum (bottom). These images are juxtaposed with Panel B, which is the topology used by SEGMEnT where crypts and villi are represented with a matrix of rectangular prisms. Each individual crypt or villus is then “unwrapped” onto a 2-dimensional grid (Panel C), on which signaling interactions, morphogen diffusion and physical cellular actions take place.

**Figure 3:**
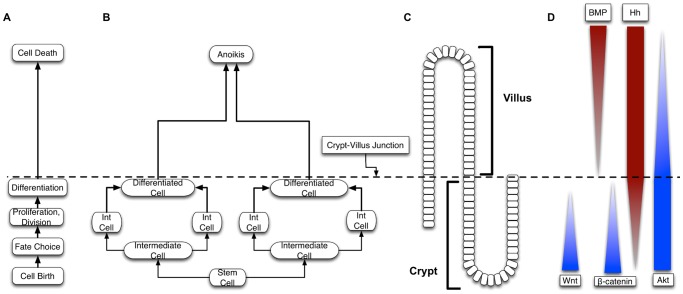
Overall schematic of SEGMEnT. Letter A depicts the cellular processes linked to Gut Epithelial Cell (GEC) type (Letter B), their spatial location in terms of the crypt-villus architecture (Letter C), and expected gradients of different morphogens (Letter D). Biologically realistic gradients for Wingless-related integration site (Wnt), Bone Morphogenetic Protein (BMP), Sonic Hedgehog Homolog (Hh), β-catenin, and Akt are shown.

SEGMEnT is a cell-level ABM: an object-oriented, discrete event, rule-based computational modeling method consisting of populations of computational entities (agents) that follow programmed rules governing their behavior with respect to the environment and interactions with other agents [19]–[24]. SEGMEnT represents the enteric mucosal surface with a 3-dimensional topology that has fixed central axes for the crypts and villi, with square grids over-laid onto an array of rectangular prisms to represent the epithelial surfaces of the crypts and villi (Figure 2). The primary cell types represented in SEGMEnT are gut epithelial cells (GECs), including their subtypes of stem cells, differentiating and mature enterocytes, and two main lineages of inflammatory cells, neutrophils and macrophages/monocytes. SEGMEnT integrates multiple functional modules including an intra-cellular morphogenic signaling pathway, an intra-cellular inflammatory signaling pathway, cell state transitions for proliferation, differentiation and movement, and spatial diffusion of morphogens to define gut epithelial cell behavior for the homeostatic maintenance of the spatial architecture of the enteric mucosa (Figure 3). Figure 3 depicts cellular processes (Letter 3A) represented, those processes linked to GEC type (Letter 3B), their spatial location in terms of the crypt-villus architecture (Letter 3C), and the spatial distribution of expected gradients of different morphogens (Wingless-related integration site (Wnt), Bone Morphogenetic Protein (BMP), Sonic Hedgehog Homolog (Hh), β-catenin, and Protein Kinase B (Akt) are shown in Letter 3D). For a comprehensive depiction and list of the molecules in the signaling networks included in SEGMEnT see Table S1 and Figure S1. More detailed information regarding network connection strengths and signaling molecule functions can be found in Tables S2 and S3. For an overview of the signaling pathways involved see Figure S1; implementation details of these pathways are presented in the Materials and Methods and Supplementary Materials Text S1. All epithelial and inflammatory cells share the same inflammatory signaling pathway structure, though with differential behaviors defined through altered signaling rates. Abstracted blood vessels are also incorporated as points of arrival for circulating inflammatory cells. SEGMEnT implements an expandable architecture that will allow the subsequent addition of functional modules, and the ability to represent additional enteric features (Figure 1B).

SEGMEnT aims to replicate the dynamically stable morphology and cellular populations of the healthy ileum, while matching the spatial distribution of molecular signaling gradients reported in the literature. SEGMEnT also reproduces the effects of inhibition of various signaling pathways by matching resultant phenotypic alterations of the crypt-villus architecture as reported in the literature. SEGMEnT is used to investigate two general types of potentially pathogenic conditions: 1) those resulting from acute insults/perturbations, namely local tissue injury resulting in a wound and the response to enteric ischemia-reperfusion, and 2) chronic conditions that result in a persistent alteration of the homeostatic set-point of the crypt-villus architecture. For this latter case we use the example of colonic metaplasia of the ileal pouch following restorative surgery for ulcerative colitis, thought to be induced by chronic, low-level inflammation due to fecal stasis [25]–[27]. In the course of model development it became apparent that the recognized links between inflammation and tissue patterning in the gut were not sufficient to generate the metaplasia phenotype. This insufficiency in the current state of knowledge led us to hypothesize a link between inflammation and the induction of apoptosis through the Phosphotase and tensin homolog/phosphoinositide 3-kinase (PTEN/PI3K) pathway, a relationship present in other tissues [28] but not previously suggested in the gut. We demonstrate that SEGMEnT, with this putative mechanism, is then able to generate colonic metaplasia of the ileum as seen after surgery for ulcerative colitis. SEGMEnT is the dynamic knowledge representation of a minimally sufficient set of components, mechanisms and interactions for the maintenance of enteric mucosal architecture (morphogenesis) and the effect of inflammation. The eventual goal for SEGMEnT is to serve as a community resource by acting as a “virtual sandbox” that allows researchers to instantiate and investigate their own knowledge, and try out novel and innovative hypothesis for a range of intestinal diseases.

## Results

Simulations with SEGMEnT fall into three categories: 1) those representing the baseline healthy state of the gut mucosa, 2) those reproducing previously published single-molecule/gene knockout/inhibition experiments, and 3) those reproducing published experiments investigating specific pathophysiological conditions. For details of the underlying biology in SEGMEnT and the development process see the Materials and Methods and the Supplementary Materials Text S1.

### Baseline healthy ileal tissue dynamics

Integral to the use of computational models as a means of integrating mechanistic knowledge is the ability of those models to replicate baseline, healthy behavior [29], [30]. Therefore, the first set of simulations calibrated SEGMEnT to generate and sustain dynamically stable configurations corresponding to baseline enteric tissue behavior. While SEGMEnT does not generate the spatial architecture de novo, it is able to produce the appropriate cell distributions and spatial morphogen gradients leading to homeostasis. The homeostatic crypt-villus configuration in the ileum consists of a crypt depth approximately 1/4 the villus height (approximately 1 mm) [31]. Spatial gradients exist for morphogens such as Wnt, which is highest at the base of the crypt and zero at and above the crypt-villus junction [32]–[37]. The BMP molecule exists everywhere in an isotropic distribution [38], while the gradient of BMP activity is initiated at the crypt-villus junction progressively maximizes towards the tip of the villus [38]–[40]. Other morphogens included in SEGMEnT, Ephrin-B ligand/EphB receptor, Hh, and β-catenin, also exhibit spatial gradients [32]. Furthermore, the cellular components of the epithelium undergo a complete renewal every five days.

**Figure 4:**
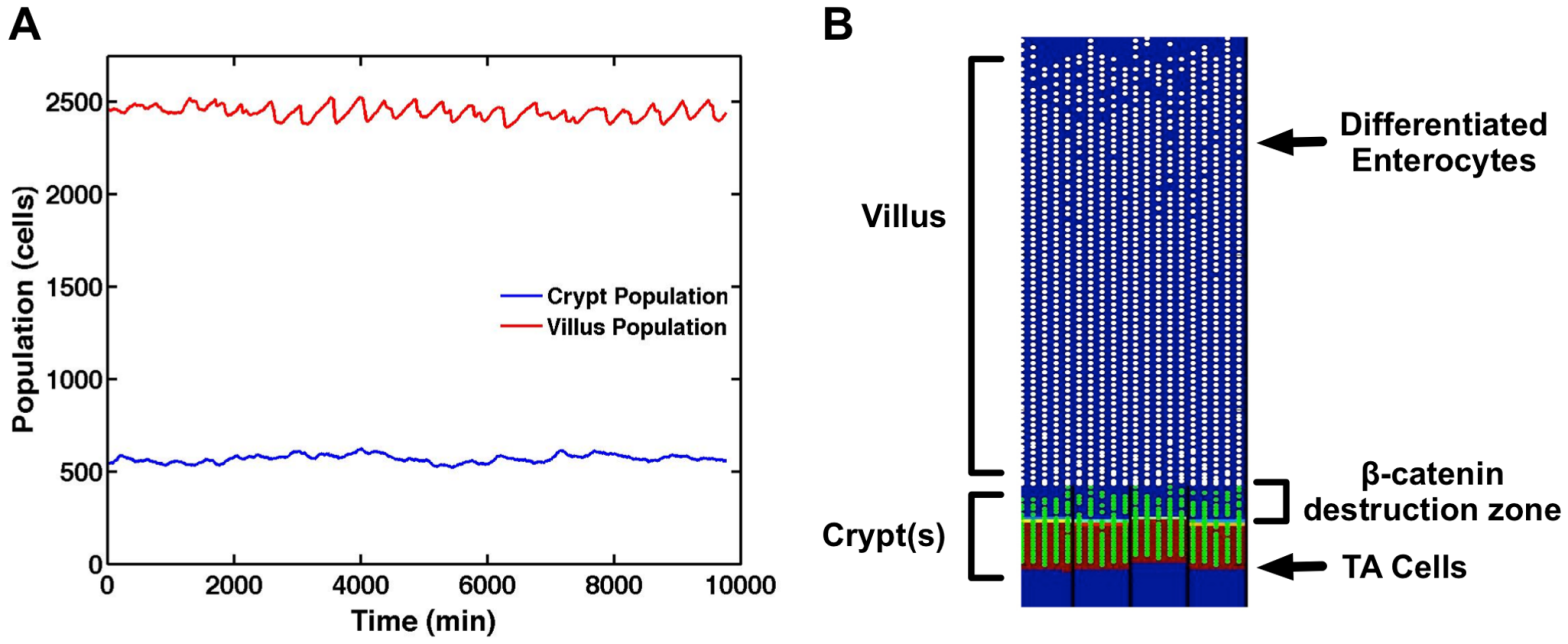
Baseline healthy ileal tissue dynamics. Panel A demonstrates stable cell populations in the crypt and villus over time with complete renewal of the epithelial tissue every 5 days of simulated time. Panel B displays a SEGMEnT screenshot at homeostasis. Note that this 2d projection is the unwrapped 3d topology seen in Figure 2. All subsequent SEGMEnT screenshots are presented in this fashion to aid in the interpretation of its behavior, but are simulated in a 3d model. Differentiating GECs in the crypts are seen as green circles; white circles represent differentiated GECs on the villi. The red shading in the background represents areas of Wingless-related integration site (Wnt) activity; the absence of Wnt is seen as a blue background. Note that at the crypt-villus junction there is a region of undifferentiated GECs with no Wnt. Since activated Wnt signaling is identified and localized by the accumulation of β-catenin, the absence of Wnt in this zone denotes a region after the activation of β-catenin destruction complex and before all the β-catenin has been destroyed, at which point differentiation is triggered and occurs. This pattern of β-catenin activity is consistent with patterns seen in published histological data seen in Figures 3a and 3c in Ref [33].

Figure 4 shows the output from a simulation for healthy baseline ileal tissue at homeostatic equilibrium. Stable cell populations in the crypt and villus are seen over time (Figure 4A) with complete renewal of the epithelial tissue every 5 days of simulated time. A SEGMEnT screenshot at homeostasis is seen in Figure 4B. At the crypt-villus junction there is a region of undifferentiated gut epithelial cells (GECs) with no Wnt. Since activated Wnt signaling is identified and localized by the accumulation of β-catenin, the absence of Wnt in this zone denotes a region after the activation of β-catenin destruction complex and before all the β-catenin has been destroyed, at which point differentiation is triggered and occurs. This pattern of β-catenin activity is consistent with patterns seen in published histological data seen in Figures 3a and 3c in Ref [33].

Figure S2 shows SEGMEnT's output in terms of both extracellular BMP and binding of BMP with its epithelial cell receptor. These simulation results can be compared with histology images from Reference [38] that demonstrate free extracellular BMP is evenly distributed throughout the tissue (Figure 1d from Ref [38]), whereas the binding of BMP with its cellular receptor, and hence its activity, is maximal in the enterocytes at the villus tip with decreasing binding down towards the crypt-villus junction (Figure 1f from Ref [38]). Output from SEGMEnT visualizing the distribution of free and bound BMP compare well with these images, where the extracellular BMP molecule concentration is roughly uniform distribution (Figure S2, Panel A, shown in blue, compare Figure 1d from Ref [38]). Simulated BMP activity/binding gradient matching experimental data can be seen in Figure S2B, with brown gradients representing bound BMP (compared to brown stained enterocytes seen in Figure 1f from Ref [38]), with corresponding areas of low BMP activity in the crypts (green brackets), initiation of BMP activity at the crypt-villus junction (yellow brackets) and high BMP activity at the tip of the villi (red brackets).

### Morphogen pathway component inhibition/knockouts

Selective pathway component knockout and/or inhibition studies are a mainstay of molecular biology[41]. The results of these types of experiments are often used to infer the mechanistic role of the targeted pathway component. From the standpoint of modeling and simulation, this approach can be viewed as model component sensitivity testing, and form a ready set of real-world experimental reference sets against which to evaluate the plausibility of a dynamic computational model. In order to provide baseline validation of SEGMEnT's implementation of enteric morphogenesis pathways, a series of simulation experiments were performed for three knockout/inhibition conditions, Wnt inhibition, Hh inhibition, and PTEN inhibition, and compared to previously published experimental data concerning the effects of these interventions. Note that we use the broad definition of “validity” as given in the recognized dictionary of modeling terms [42], namely in terms of establishing the believability or trust in a particular model by reproducing sets of identified behaviors in the real world referent.

**Figure 5:**
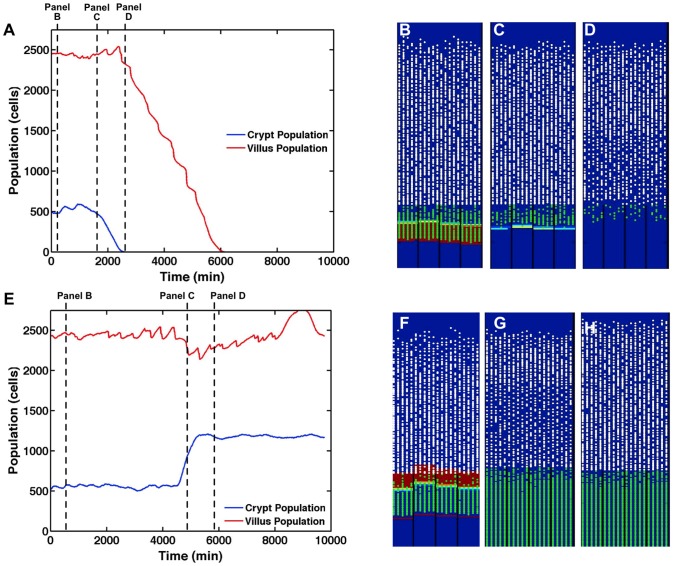
Morphogen knockout/inhibition simulations. Panel A displays average crypt and villus GEC populations in SEGMEnT when exposed to Wingless-related integration site (Wnt) inhibition. Panels B–D displays three frames from a SEGMEnT simulation of Wnt inhibition. Panel B represents baseline homeostasis prior to Wnt application. Panel C was captured at t = 1755 min, shortly after Wnt application. Cells then quickly differentiate as the β-catenin destruction complex is activated; the increased Hh signal from the newly differentiated cells combined with the Wnt inhibitor quickly eliminate all Wnt activity. Panel D was captured at t = 2160 min, at which point with the elimination of Wnt, the crypt no longer exists, there are few proliferating cells, and the villi have lost significant cellularity. Panel E displays average crypt and villus GEC populations in SEGMEnT when exposed to Sonic Hedgehog Homolog (Hh) inhibition. Panel F represents the system at baseline homeostasis prior the application of Hh inhibition. Panel G was captured at 5010 min, shortly after application of Hh inhibition. After application of the Hh inhibitor, the crypt expands rapidly. Initially, there is slight villus shrinkage as differentiation is temporarily halted by the now uninhibited Wingless-related integration site (Wnt) gradient. Panel H was captured at t = 6000 min. As the system adjusts to the lack of Hh, homeostasis is reached and persistent crypt hyperplasia exists throughout the system (Panel H).

The Wnt pathway is a fundamental pathway to gut morphology, and the consequences of its inhibition are well documented [32], [34]–[36]. When healthy ileal tissue is exposed to a strong Wnt inhibitor, the system exhibits a loss of proliferating cells, followed by a loss of crypts, followed by total organ failure and death [32], [34]–[36]. A Wnt inhibitor is applied to the system after it evolves to a steady state, and SEGMEnT produces results that match experimental observations (Figure 5). Figure 5A shows the average population of differentiated and undifferentiated cells (which can be considered a proxy for crypt depth and villus height respectively) vs. time, with the initial loss of crypt cells followed subsequently by loss of villus cells and complete population collapse. Figure 5B–D displays three frames from a SEGMEnT simulation showing the temporal change in the spatial organization of the crypt-villus architecture from baseline homeostasis (Figure 5B) to shortly after administration of the Wnt inhibitor, which leads to a decrease in the crypt population (Figure 5C). The loss of proliferating cells percolates to the villus populations (Figure 5D), preceding the death of the entire villus. These behaviors correspond to the outcomes reported in the literature concerning the effect of Wnt inhibition in producing first crypt-villus atrophy and then death [32], [34]–[36].

Computational models provide detail into the progression of events; here, one can observe that gut epithelial cells quickly differentiate as the β-catenin destruction complex is activated. The increased Hh signal from the newly differentiated cells leads to the production of the Wnt inhibitor, secreted frizzled-related protein 1 (SFRP1), which quickly eliminates all Wnt activity. At this point, the crypt no longer exists, there are few proliferating cells, and the villi have lost significant cellularity; within two days, the last of the differentiated villus cells will have undergone apoptosis and the tissue is dead. Unfortunately, at this time, quantitative experimental data characterizing the dynamic properties of the respective cell populations and their respective molecular signatures is not available, but qualitatively, the output from SEGMEnT matches that seen in experiment in terms of crypt-villus atrophy and subsequent death [32], [34]–[36].

Hh inhibition, while less studied, represents the diametrically opposed case to the Wnt pathway [43], [44]. Experimentally, the inhibition of Hh signaling in the ileum results in increased Wnt activity, crypt hyperplasia, and crypt fission [45]. When an Hh inhibitor is applied to the model at homeostasis, the system's SFRP1 is quickly depleted, allowing the Wnt source to operate unopposed. As Wnt activity increases, the villus shrinks slightly and the crypt displays significant growth (Figure 5E). Once the crypt has reached a stable equilibrium with regards to the new Wnt gradient (unaffected by Hh), the villus grows back to a normal size, leaving us with a normal villus and a deeper than normal crypt (Figure 5H). When simulating Hh inhibition, SEGMEnT generates increased Wnt activity and crypt hyperplasia as reported in the literature [43], [44]. Crypt fission is currently beyond the scope of this work.

While experimental inhibition of PTEN does not change epithelial architecture (in the long term) from the normal state in the absence of inflammation [46], some observations suggest the putative down-regulation of Hh by PTEN [39]. Additional studies suggest that Hh is down regulated by inflammation [37]; therefore we posit that it is the up-regulated PTEN that is responsible for the down-regulated Hh, with subsequent effect on GEC apoptosis.

**Figure 6:**
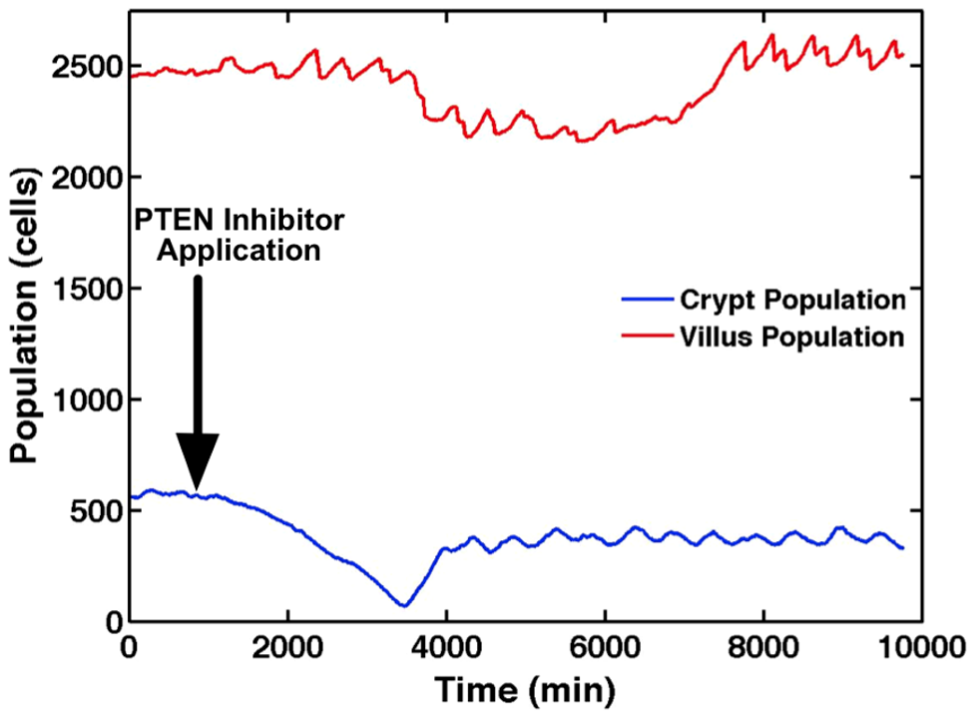
PTEN inhibition. Average crypt and villus populations are presented when exposed to Phosphotase and tensin homolog (PTEN) inhibition. Immediately subsequent to the PTEN inhibition (Arrow), levels of Sonic Hedgehog Homolog (Hh) spike, resulting in an immediate shrinkage in the crypt. With Wingless-related integration site (Wnt) inhibited by the higher levels of Hh, cell proliferation slows, resulting in slight villus atrophy. The system then reaches a new equilibrium absent PTEN via these redundant pathways, resulting in essentially the baseline tissue architecture. It is notable that the compensatory dynamics seen in the simulation experiments occur in a period not captured by the sampling interval in [46].

Simulation of PTEN inhibition demonstrates a “null” consequence of SEGMEnT's PTEN representation on baseline morphogenesis; we hypothesize that its role would only become significant in the perturbation case involving inflammation. Figure 6 displays output from SEGMEnT when PTEN has been strongly inhibited. SEGMEnT simulations of unperturbed tissue show Hh and PTEN sharing the responsibilities of apoptotic regulation and villus maintenance. When Hh activity is inhibited, PTEN takes over; when PTEN in inhibited, the Hh pathway assumes these responsibilities. The simulation experiments demonstrate this transient effect on the crypt GEC population and which subsequently recovers with compensation via Hh. The resulting crypt villus architecture at the end of 24 hrs appears relatively normal, consistent with experimental findings [46]. It is notable that the compensatory dynamics seen in the simulation experiments occur in a period not captured by the sampling interval in Ref [46]. While the end result of a SEGMEnT simulation of PTEN inhibition closely matches experiment, further experiments are necessary to elucidate the progression of events from PTEN inhibition to the establishment of a new equilibrium, which happens to be similar to the PTEN active equilibrium.

### Local mucosal wound

All organisms have evolved in a heterogeneous and potentially dangerous environment and thus have to be able to deal with injury in order to maintain their bodily integrity. The inflammatory response is a highly conserved system that represents an organism's initial active response to these threats, providing initial containment and control and prompting the subsequent healing process [47], [48]. This function physiologically manifests in response to local tissue damage level as successful healing, and the expectation is that a model of normal tissue should be able to accomplish successful wound healing up to a certain level of injury. There is extensive literature concerning the computational modeling of wound healing, primarily in terms of epithelial cell dynamics and migration [49]–[51], though application to intestinal injury and healing is limited to necrotizing enterocolitis. However, even though they are not specific to the gut, the models of epithelial injury and recovery do share many of the same pathways concerning inflammation with SEGMEnT.

**Figure 7:**
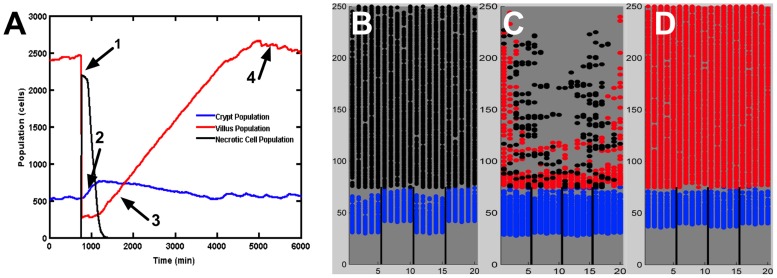
Local injury to the gut mucosa and subsequent reconstitution of crypt-villus architecture. Panel A displays populations of living gut epithelial cells (GECs) on the crypt and villus, and necrotic cells upon local tissue injury and subsequent healing. Injury is induced by causing necrosis of an entire villus, resulting in a rapid drop of villus GEC population and spike in necrotic cell population (Arrow 1). In the time period directly subsequent to villus death the crypt grows rapidly; this is due to the sudden loss of Sonic Hedgehog Homolog (Hh) signaling as most of the differentiated cells on the villus have died. The death of the villi cells reduces the Wnt inhibition of the surviving crypt GECs, resulting in a growth spike in the crypt population (Arrow 2) that precedes the reconstitution of the villus population (Arrow 3). All during this process the inflammatory response is clearing the necrotic cells, allowing the regulatory functions of the morphogen pathways to normalize, leading to regrowth of the villus back to the homeostatic state (Arrow 4). Panels B–D display three screenshots from a simulation of localized epithelial damage/injury. Undifferentiated transit amplifying (TA) cells are shaded in blue. Differentiated enterocytes are shaded in red. Necrotic cells are shaded in black. Panel B, captured at t = 750 min, demonstrates that the epithelial insult results in necrotic cell death throughout the majority of the villus. Panel C, captured at t = 2250 min, shows that the necrotic cells continue to damage surrounding tissue until clearance by macrophages and neutrophils (not explicitly represented in these screenshots). Panel D, captured at t = 4500 min, demonstrates the recovered tissue architecture after regrowth of the epithelial cell populations.

SEGMEnT simulations of localized epithelial injury demonstrate the ability of the simulated tissue to heal itself after the infliction of a localized wound to the mucosa. A tissue wound/injury is represented by activating sufficient toll-like receptor (TLR4) signaling to cause all cells on the villi to die simultaneously by necrosis (Figure 7A Arrow 1). In the time period directly subsequent to villus death the crypt grows rapidly; this is due to the sudden loss of Hh signaling as most of the differentiated cells on the villus have died. The death of the villi cells reduces the Wnt inhibition of the surviving crypt GECs, resulting in a growth spike in the crypt population (Figure 7A, Arrow 2). As these undifferentiated GEC migrate back up the crypt they lead to the reconstitution of the villus (Figure 7A, Arrow 3). The inflammatory response to the tissue damage clears the necrotic cells (see Figure 7B–D below) and allows restoration of the regulatory functions of the morphogen pathways, with subsequent regrowth of the villus back to the homeostatic state (7A, Arrow 4). Figure 7, Panels B–D display three screenshots of output from the SEGMEnT during this process: Panel B depicts the period immediately following the injury, with necrotic cells seen in place before they are cleared. In Figure 7 Panel C inflammatory cells begin to infiltrate the tissue, both from the activation of local inactive monocytes and the arrival of active neutrophils and macrophages from local blood vessels. These inflammatory cells follow gradients of interferon-γ (IFN-γ) and TLR4 ligand, respectively. The inflammatory cells clear out the necrotic debris via phagocytosis, halting further damage signaling from the necrotic debris. Once sufficient debris has been cleared, the villus begins to repair itself at a regular rate. Figure 7 Panel D depicts 24 hours post-injury, by which time necrotic debris is completely cleared, allowing for the epithelium to quickly return to homeostasis.

### Intestinal ischemia/reperfusion (I/R)

Intestinal ischemia/reperfusion (I/R) is a significant clinical pathophysiological entity. When an individual is under extreme stress, e.g. severe injury, blood loss or shock, the body's response is to divert blood flow away from the enteric circulation towards the more immediately critical organs such as the heart and brain, sacrificing intestinal ischemia for immediate survival. Additionally, certain types of major thoracic or abdominal surgery require a temporary occlusion of intestinal blood flow, leading to a period of enteric ischemia. The reperfusion phase involves a rapid influx of blood and circulating immune/inflammatory cells into an endothelial surface that has been primed by the ischemic period, which results in an acute activation of the inflammatory response [52]. This leads to a dramatic change in the enteric mucosal tissue, with induction of tissue inflammation, cellular death and turnover [53], and also affecting resident gut microbes [54]. Reperfusion also leads to sloughing/shedding of ischemic cells into the intestinal lumen). It should be noted that I/R-induced enterocyte sloughing represents a relatively unique means of eliminating ischemic and impending necrotic cells: as opposed to clearance of necrotic cells via enzymatic degradation and phagocytosis as in most tissues, I/R induced enterocyte sloughing takes advantage of the spatial fact that there is a place (i.e. the intestinal lumen) where these potentially necrotic cells can be eliminated. It has been suggested by other researchers that this mechanism for removing ischemic enterocytes, thereby reducing the forward feedback loop propagating inflammation, represents a protective mechanism against I/R of the small intestine [55], [56]. While the specific mechanisms for I/R-induced sloughing are still under investigation, one proposed hypothesis involves the accumulation of Intestinal Fatty Acid Binding Protein (I-FABP) in ischemic cells and its role in promoting sloughing at the onset of reperfusion [55]. We implemented this mechanism into SEGMEnT and then tested the conceptual basis of the hypothesis that sloughing was a protective mechanism by simulating the contra-factual condition by artificially reducing the sloughing rate of the GECs and measuring its effect on the degree of crypt/villus injury (Section 2.4.2).

#### Tissue response to increasing periods of ischemia with GEC sloughing

**Figure 8:**
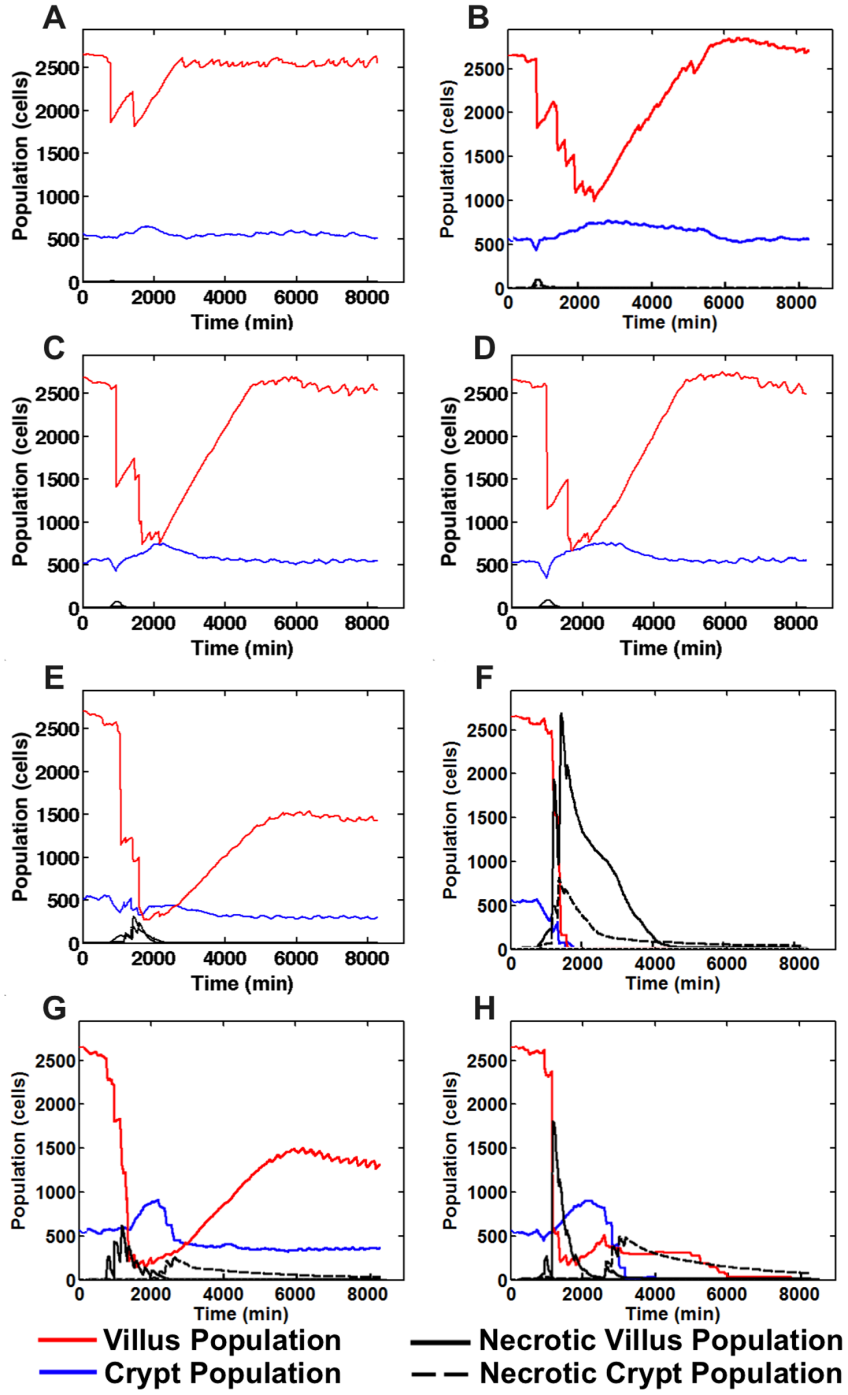
Normal and aberrant tissue response ischemia/reperfusion across a range of ischemic times. With 30 minutes of ischemia (Panel A) there is minimal necrosis and the system returns to normal by ∼24 hrs. Increasing amounts of ischemia at 2 hrs (Panel B), 3 hrs (Panel C) and 4 hrs (Panel D) all demonstrate recoverable tissue architecture. With 5 hrs ischemia (Panel E) there is a persistent alteration in the tissue composition. Application of 6 hrs ischemia (Panel F) results in a non-recoverable injury. Panels G and H show corresponding behavior in the absence of sloughing (Panel G = 30 min ischemia, Panel H = 3 hrs ischemia). The lack of sloughing increases the number of necrotic cells in place, leading to increased inflammatory stimuli and the propagation of inflammation-mediated damage, converting readily recoverable ischemia at 30 minutes and 3 hrs to correspondingly persistent effects on the tissue architecture at 30 minutes ischemia (Panel G) and tissue necrosis with 3 hrs of ischemia (Panel H).

Figure 8 displays the output of SEGMEnT simulations across a range of ischemic periods from 30 minutes to 6 hours, followed by reperfusion. In 30 minutes of ischemia (Figure 8A) the upper portion of the villus, subjected to the highest degree of ischemia, sloughs post-reperfusion, reducing the amount of potential pro-inflammatory signals and limiting inflammatory cell infiltration. As a result there is minimal necrosis and the system returns to normal by ∼24 hrs. This behavior closely matches results reported by Derikx et al [55]. Increasing amounts of ischemia at 2 hrs (Figure 8B), 3 hrs (Figure 8C) and 4 hrs (Figure 8D) all demonstrate recoverable tissue architecture, with transient decreases in villus cell populations due to sloughing. By 5 hrs ischemia (Figure 8E) the protective effect of sloughing is insufficient to prevent a persistent alteration in the tissue composition, where the number of necrotic cells rises more sharply, resulting in the death of ISCs at the base of the crypt and affecting the recoverability of the system. Application of 6 hrs ischemia (Figure 8F) results in a non-recoverable injury, with necrosis widely present in both villus and crypt, propagating inflammation and leading to viable crypt and villus populations dropping to zero. These behaviors are consistent with the recognized response of the gut to ischemia reperfusion injury [52], [53].

#### Lack of GEC sloughing worsens inflammatory damage and recovery

We test the hypothesis that GEC sloughing is protective to I/R injury by performing simulations where FABP-mediated sloughing is deactivated (Figure 8G–J). The lack of sloughing increases the number of necrotic cells in place, leading to increased inflammatory stimuli and the propagation of inflammation-mediated damage. This phenomenon is seen in the conversion of readily recoverable ischemia at 30 minutes and 3 hrs (Figures 8G and H), to correspondingly persistent effects on the tissue architecture at 30 minutes ischemia (Figure 8I) and tissue necrosis with 3 hrs of ischemia (Figure 8J). These results reinforce the plausibility that sloughing has a protective effect [55]. Note that there is no current experimental data with which to compare the non-sloughing simulations; however these results describe target conditions that may guide future potential laboratory experiments.

### Colonic metaplasia of the ileum: A hypothesized role for PTEN

Metaplasia is the reversible transformation of one type of tissue architecture into one resembling another type of tissue. It is distinct from neoplasia or dysplasia insomuch that the cells themselves do not exhibit dysfunctional growth but rather alter their differentiation path towards a different terminal cellular phenotype. Metaplasia is reflective of an alteration of the tissue environment that subsequently favors the new cellular phenotype, and is therefore often seen in chronic disease states. One example of this is seen in the small intestine following definitive surgery for ulcerative colitis resulting in the creation of an “ileal pouch:” after removal of the colon and rectum, the rectal vault is reconstructed by creating a looped pouch of the terminal ileum. This ileal pouch then serves as a neo-reservoir for the stool in an attempt to more closely approximate normal bowel habits for these patients. However, this process now changes the tissue environment for the ileal mucosa in the pouch. Even though the composition of the intestinal contents entering the pouch may not be considerably different than before, now the previously transitive luminal contents are subject to stasis, which results in an alteration of the environment for the ileal mucosa and can result in *colonic metaplasia* of the pouch epithelial tissue [25]–[27]. While not a true conversion to colonic tissue, the metaplastic epithelial architecture exhibits defined changes that more closely resemble colonic tissue: a change in the crypt-villus relationship where the crypts deepen and the villi become shortened and an increase in the relative population of goblet cells (mucous producing cells) [25]–[27].

Chronic, low-level inflammation has been associated with colonic metaplasia, and has been implicated as a mechanism driving the alterations seen in the mucosal architecture [27]. However, previously identified connections between inflammatory signaling and the morphogenesis pathway [57]–[62] did not produce the appropriate tissue architecture alterations, i.e. increasing crypt depth and shortened villi, consistent with the metaplasia phenotype. To address this issue we identified the key role of apoptosis (programmed cell death) in the generation of the metaplasia architecture: apoptosis plays a crucial role in morphogenesis by regulating life span of the GECs, subsequently affecting the height of the villus. However, existing knowledge links inflammation primarily to either anti-apoptotic (i.e., NFκB) or necrotic behavior (i.e. Receptor Interacting Protein Kinase, or RIP), neither of which would generate or is associated with a colonic metaplasia phenotype in the ileal pouch. Therefore, there existed a gap between the recognized role of inflammation and the actual processes needed to generate the target phenotype. A search of the literature identified that one proposed link between inflammation and the induction of apoptosis is through the Phosphotase and tensin homolog/phosphoinositide 3-kinase (PTEN/PI3K) pathway [28]. Based on this report, we hypothesized a putative link between gut epithelial inflammation and its effect on enteric mucosal tissue patterning: GEC apoptosis induced via the PTEN/PI3K pathway (Figure S1). The incorporation of this hypothetical mechanism not only increases the rate of GEC apoptosis, thus shortening the villus, but also inhibits Hh production. Inhibition of Hh production leads to reduced inhibition on the Wnt pathway and increases the size of the proliferative compartment in the crypt, thereby generating the essential crypt-villus architectural features characteristic of colonic metaplasia.

**Figure 9:**
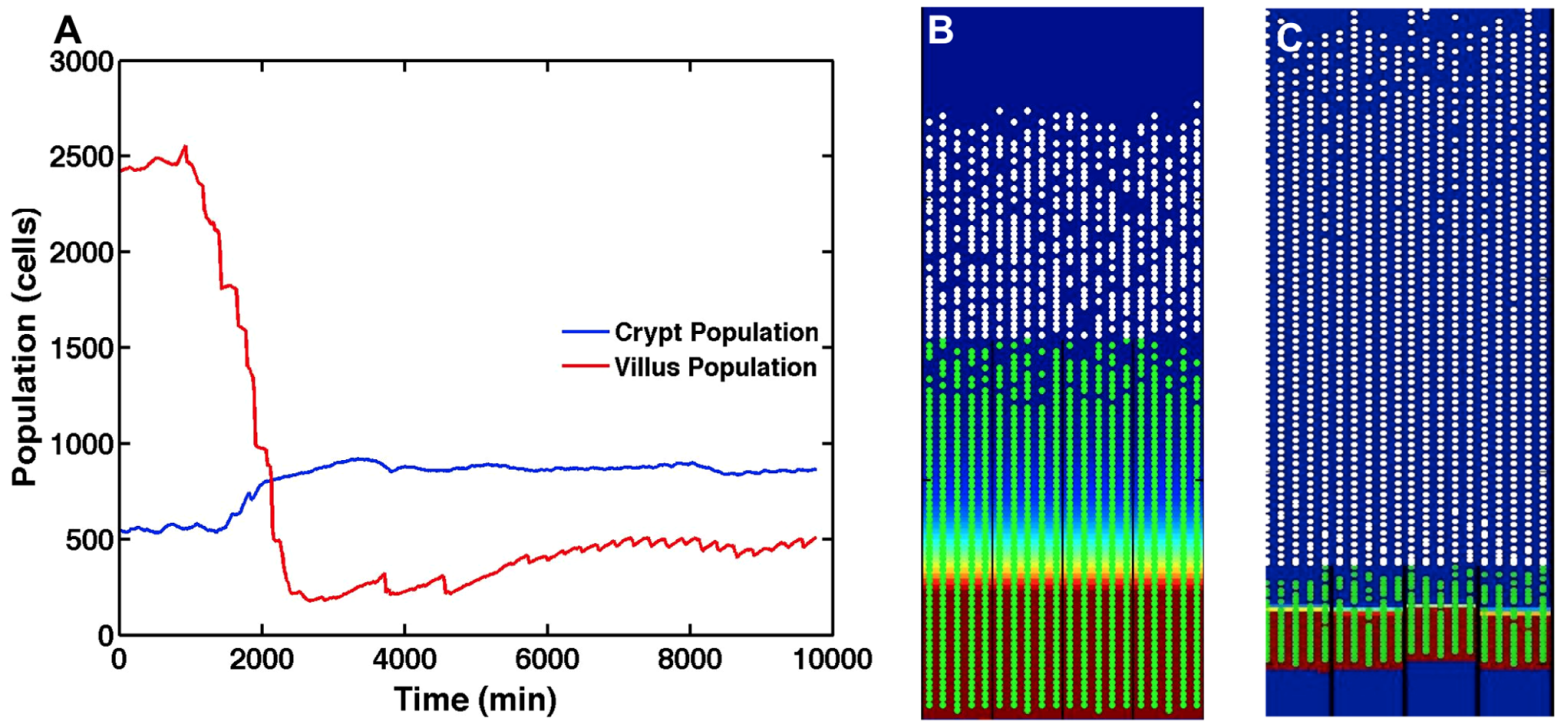
Colonic metaplasia in the ileal pouch. Panel A displays average crypt and villus gut epithelial cell (GEC) populations after exposure to sustained low-level toll-like receptor (TLR4) stimulation and signaling (an abstraction of fecal stasis). This low-level up-regulation of inflammation communicates via our hypothesized Phosphotase and tensin homolog (PTEN) mechanisms, leading to increased apoptosis, shortening the villus, as well as an inhibition of the Sonic Hedgehog homolog (Hh) pathway, which increases the size of the proliferative compartment (i.e. crypt). Panel B displays a screenshot from SEGMEnT when simulating conditions leading to colonic metaplasia. Crypt hyperplasia and villus atrophy are clearly evident (compare with normal homeostatic condition in Panel C, and as seen in Figure 4C), along with a shift in the villus to crypt height ratio that matches the alterations seen in colonic metaplasia as reported in Ref [27].

Simulations were performed with SEGMEnT to demonstrate the plausibility of a hypothesis previously published in the literature that prolonged low-level inflammation, acting as a persistent perturbation to the signaling network, would lead to a change in the morphology of the epithelial layer [27]. The simulation experiments involved implementing a continuous low-grade stimulation of TLR4s on SEGMEnT's GECs to represent a chronic low-level inflammatory milieu, mimicking the effects of luminal stasis and bacterial overgrowth in an ileal pouch. The effect of this condition on the crypt/villus architecture was evaluated in terms of alterations of the crypt/villus ratio as well as absolute changes in both crypt and villus dimensions. Figure 9A displays crypt and villus GEC populations when the system is exposed to chronic low-level TLR4 signaling (an abstraction of fecal stasis). This up-regulation leads to an increased rate of apoptosis, shortening the villus, as well as an inhibition of the Hh pathway, which leads to an increase in the size of the proliferative compartment. Figure 9B displays output from SEGMEnT when simulating conditions leading to colonic metaplasia. Crypt hyperplasia and villus atrophy are clearly evident (compare with normal homeostatic condition in Figure 9C, and as seen in Figure 5C), along with a villus to crypt height ratio that matches the alterations seen in colonic metaplasia [27], suggesting the plausibility of this mechanism as the driver for colonic metaplasia.

## Discussion

SEGMEnT dynamically represents and integrates existing knowledge concerning homeostasis and inflammation in the ileum and provides a computational platform to augment the exploration of the cellular/molecular processes involved in intestinal wound repair, ischemia/reperfusion injury, and colonic metaplasia/pouchitis. SEGMEnT successfully replicates the dynamically stable morphology and cellular populations of the healthy ileum, while qualitatively matching the spatial distribution of molecular signaling gradients consisting with the existing qualitative histological criteria utilized and reported in the literature [32]–[38], [40]. SEGMEnT also reproduces the effects of inhibition of various signaling pathways by successfully representing the resulting phenotypes in terms of alterations of the crypt-villus architecture [32]–[38], [40]. With the integration of inflammation as a control input to the morphogenic signaling pathway, SEGMEnT demonstrates the ability to withstand certain acute perturbations (local tissue injury, ischemia/reperfusion) as would be expected from normal intestinal tissue, as well as reproduce a specific chronic pathological state associated with inflammatory stimulation (colonic metaplasia). Furthermore, simulations reinforce the protective role of enterocyte sloughing in enteric ischemia-reperfusion and demonstrate the plausibility of PTEN as a cross-talk nexus between inflammation and epithelial patterning in the continuum effect of inflammation on the genesis of colonic metaplasia. Our hypothesis of the role of PTEN in the generation of metaplasia is an example of the type of exploratory modeling that could be performed using computational tools such as SEGMEnT. Further exploration of this concept could follow several investigatory paths. From a cell culture/cell biology standpoint, enteroid preparations could be evaluated under various types of inflammatory cytokines/simulation for levels of PTEN expression, corresponding downstream mediator expression and rates of apoptosis. From an in vivo perspective, a potential animal model of metaplasia and pouchitis should demonstrate an up-regulation of PTEN corresponding to greater histological changes, and the application of a local PTEN inhibitor should reverse or block the generation of metaplasia. Finally, clinical investigations could take a similar cohort to Ref [26] (two groups of patients with an ileal pouch – one that obtained the pouch as a result of ulcerative colitis and one that received the pouch as a result of familial adenomatous polyposis) and perform gene expression analysis on tissue collected from the ileal pouch.

SEGMEnT follows in a line of prior spatially explicit computational models investigating the dynamics of cell populations and signaling within the intestinal crypts [14]–[18]. The Meineke model represents an early attempt to characterize crypt cell population distributions in a spatial context, using an abstracted fixed cylindrical topology and an off-lattice, Voronoi tessellation to represent continuous cell movement, but did not include any representation of cell signaling [16]. Murray et al investigated cell transit between colonic crypts arising from various perturbations to a simulated Wnt pathway [14]. The Van Leeuwen model examines how the Wnt pathway and differences in its signaling progression might affect epithelial cell proliferation [15]. Buske et al investigated the dynamics of cell-fate determination under different conditions and how maintenance of cellular organization could proceed without the need to invoke stem cell behavior [17]. Wong et al utilized a cellular Potts model to investigate the role of EphB/Ephrin-B on cell migration velocities and trajectories within the crypt [18]. Pin, et al use a 3-dimensional Monte Carlo model with a fixed spiral architecture to simulate the dynamics of intestinal epithelial migration and their work includes simulations of crypt cell population regeneration following injury approximating radiation-induced damage, though the response to injury in this model is limited to the morphogenic processes associated with crypt repopulation and does not include inflammatory factors [63]. There have also been several projects involving computational modeling of intestinal inflammation and injury, focusing on intestinal host-microbe effects [64]–[66], the effects of sepsis on the gut [67], or on a specific disease process, namely necrotizing enterocolitis [68]–[71], or inflammatory bowel disease [72]. While none of these prior models have attempted to examine the specific tissue architectural consequences of inflammation, nearly all of them incorporate the same general set of inflammatory pathways seen in SEGMEnT.

SEGMEnT incorporates and integrates the features seen in the spatial gut models concerning the Wnt/Notch pathways with the inflammatory components and processes seen in the gut inflammation models. As such, it is able to extend the scope of its representation to investigate how inter-pathway specific interactions can affect other pathways in the overall signaling network. This capability allowed the instantiation of a novel hypothesis concerning a link between inflammation and morphogenesis through the PTEN/PI3K pathway. Additionally, prior spatial gut models all utilized a fixed crypt topology (i.e. height/depth) upon which their cellular components moved, and therefore are not able to examine metaplasia, ischemia/reperfusion or injury and repair, all conditions that result in some distortion of the baseline crypt-villus architecture. SEGMEnT expands on these prior approaches by allowing for dynamic alteration of these critical histological dimensions; in this fashion it crosses over into some of the capabilities represented in the other arm of spatial crypt-villus modeling that is more focused on looking at factors that actually generate the tissue topology [10]–[13]. The ability to modify the dimensions of the crypt-villus complex is particularly important in being able to represent the differential tissue configurations seen in the Wnt and Hh knockouts and colonic metaplasia.

SEGMEnT also allows the representation of multiple crypt-villus complexes and their associated external tissue structures, simulating significantly larger sections of intestinal tissue. The benefits of this have already been demonstrated in the simulations of healing local tissue injury, where a damaged villus is represented as being adjacent to the uninjured tissue involved in affecting it repair. The current version of SEGMEnT can represent up to 1.4 mm^2^ area of tissue on a single processor with 2 GB of memory. Capturing the healing of surgical anastomosis or the “patchiness” of inflammatory bowel exacerbations or environmental enteropathy will require the representation of substantially larger surface area of the gut; as such, current work is being done to implement SEGMEnT HPC to investigate the scaling issues associated with simulating physiologically/anatomically relevant areas and volumes of enteric tissue. SEGMEnT HPC is designed to represent arbitrarily large and customizable epithelial surfaces.

SEGMEnT is a work in progress, representing an instantiation of a minimally sufficient set of components and interactions to achieve a face valid representation of the mechanisms involved in maintaining the enteric mucosal architecture (morphogenesis) and its response to an initial set of perturbations (inflammation). The modular control organization of SEGMEnT (Figure 1) has been designed with expansion in mind; in Figure 1B we have positioned some of the potential avenues for development as additional control modules within this framework. Table S4 lists a set of recognized current limitations that have all been targeted for future development via the iterative refinement development process described by Hunt et al [7], [73], [74], in which additional detail is added only when new reference system features are desired, with subsequent confirmation that prior calibration/validation metrics continue to be met. This approach emphasizes the utilization of parsimonious yet functionally useful levels of abstraction and detail, providing a check to the tendency to incorporate as much detail as possible. Our eventual goal is for SEGMEnT, in its multiple forms, can serve as a community resource by acting as a “virtual sandbox” that can be used by different researchers to instantiate and investigate their own knowledge, try out novel and innovative hypotheses, and, hopefully, communicate their insights and findings to the wider community. With this in mind, work is progressing on integrating the SEGMEnT development process with our Computational Modeling Assistant (CMA) [75], a cyberworkspace that is intended to augment the ability of bioresearchers to construct and modify dynamic computational models and run and analyze simulation experiments. By linking SEGMEnT with the CMA we hope to accelerate the Scientific Cycle for the study of intestinal diseases, and aid the gastrointestinal research community in being able to overcome the Translational Dilemma [76], [77].

## Materials and Methods

SEGMEnT is implemented using the agent-based modeling software, Repast Simphony 2.0 developed at Argonne National Laboratory [78]. Construction and calibration of SEGMEnT involved assembling agent rule sets based on a review of the literature, implementation of those rules and agents within the model topology, and adjusting the components such that it could generate a dynamically stable ileal crypt-villus architecture of plausible dimensions, defined as the ratio of crypt-to-villus as equal to 1/4 [31]. It should be noted that SEGMEnT is not designed to generate the mucosal tissue architecture de novo, i.e. it is not a model of embryogenesis or organogenesis, nor is it intended to recreate the physiomechanical forces involved in determining why the architecture has the shape it does. Rather, SEGMEnT starts with the assumption that these processes have already occurred to produce an existing tissue architecture that is subsequently maintained by the behavior of the gut epithelial cells (GECs). Chemokine/mediator dynamics were tuned to create biologically realistic morphogen gradients and maintain constant cellular migration velocities. Cellular proliferation rates were adjusted so that biologically plausible populations of differentiated and undifferentiated cells are maintained when the system is in homeostasis. The list of parameters present at the end of the calibration phase can be seen in Table S1.

SEGMEnT is a knowledge-based model incorporating the inter-relationships between the various cellular and molecular entities present in the enteric tissue. Parameters for these relationships were extracted from the literature, when possible; fitting emphasized the relational qualities between processes. The multi-scale nature of the SEGMENT is apparent in the hierarchical ordering of events and components. Molecular events (occurring on the order of 10^−8^ m) are aggregated by cells (spanning 10^−6^ m), which interact to form tissue (10^−3^ m^2^). This is also true with respect to temporal scales, where the time scales on which SEGMEnT operates span several orders of magnitude. Signaling events (i.e., the binding of a ligand to a receptor) occur on a time scale from approximately 10^−3^ s to 1 s, while it can take ∼10^3^ s for the consequences of that event (the secretion of a protein) to manifest. An individual cell performs physical actions (i.e., migration, division) approximately every 10^3^ s, and ultimately survives for ∼10^4^ s. SEGMEnT then makes the implicit assumption in the time it takes for a cell to perform a physical action, any signaling events that would occur in the region of space which the cell occupies will have already occurred; thus, simple time-delayed rules give similar results to a differential equation based model, but at a fraction of the computational cost. Explicit details regarding the underlying biology and modeling methodology can be found in the Supplementary Materials Text S1.

## Supporting Information

Figure S1 **Signaling networks instantiated in SEGMEnT.** Morphogen signaling pathway components are shaded in blue; inflammatory signaling components are shaded in orange. Stimulation/production relationships are depicted by green connectors; inhibitory relationships are seen as red connectors. The signaling network comprises the Wingless-related integration site (Wnt), Bone Morphogenetic Protein (BMP), Phosphotase and tensin homolog/phosphoinositide 3-kinase (PTEN/PI3K), Sonic Hedgehog Homolog (Hh), Tumor Necrosis Factor (TNF)-α, Interferon (IFN)-γ, RIP Kinase, nuclear factor kappa-light-chain-enhancer of activated B cells (NF-κB), Janus Kinase (JAK), Signal transducer and activator of transcription 3 (Stat3), and reactive oxygen species (ROSs), and Interleukin (IL) 6,10,13, and 15 signaling pathways. Full details of the implementation of these signaling relationships are presented in the *Materials and Methods.*
(TIF)

Figure S2 **Simulation distributions of free Bone Morphogenetic Protein (BMP) and BMP binding to epithelial cells.** Panel A shows the distribution of free molecular Bone Morphogenetic Protein (BMP) concentrations in SEGMEnT: free BMP is shown as blue, and having a uniform density throughout the tissue. This corresponds to histological data showing a uniform density of free BMP from the bottom of the crypt to the top of the villus (see Figure 1d from Ref [38] for a corresponding even distribution of blue-stained free BMP). Panel B displays the BMP activity gradient, representing binding of BMP with its receptor, generated by SEGMEnT, color coded as brown to match the histological staining in Figure 1f from Ref [38]. Zones of BMP activity closely match the pattern seen in published experiments (see Figure1f from Ref [38]) with simulated BMP activity/binding gradient matching experimental data with minimal BMP activity in the crypts (green brackets), a transition zone with BMP activity beginning at the crypt-villus junction (yellow brackets) and increasing until it maximizes at the tip of the villus (red brackets).
(TIF)

Table S1 **Primary molecular components included in SEGMEnT.**
(PDF)

Table S2 **Rules for molecular interactions in SEGMEnT.**
(PDF)

Table S3 **Physical characteristics, published and derived time delays, and model-implemented time delays for signaling events involving molecular components included in SEGMEnT.**
(PDF)

Table S4 **Additional features and capabilities to be added to SEGMEnT.**
(PDF)

Text S1 **Detailed description of underlying biology and its implementation in SEGMEnT.** These materials provide a detailed description of the biological processes, modeling assumptions and implementation of the biological knowledge into SEGMEnT. These materials also provide descriptions of how the simulation experiments were performed.
(PDF)
