# Supplementary figures and images for: Investigation of Inflammation and Tissue Patterning in the Gut Using a Spatially Explicit General-Purpose Model of Enteric Tissue (SEGMEnT)
Source: PLoS Comput Biol. 2014 Mar 27;10(3):e1003507. doi: 10.1371/journal.pcbi.1003507 (PMC3967920; doi:10.1371/journal.pcbi.1003507)

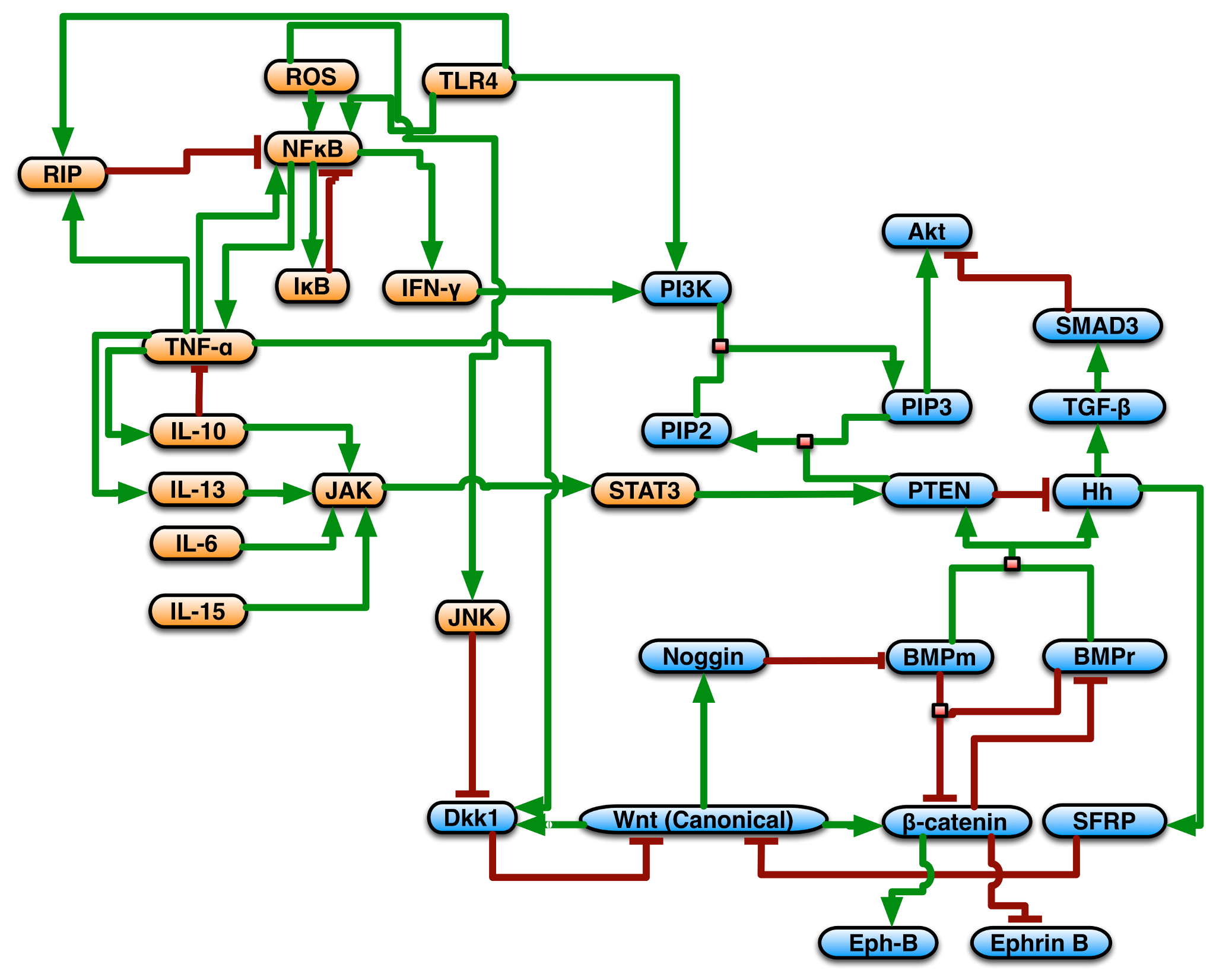

Supplement: Figure S1 — Signaling networks instantiated in SEGMEnT. Morphogen signaling pathway components are shaded in blue; inflammatory signaling components are shaded in orange. Stimulation/production relationships are depicted by green connectors; inhibitory relationships are seen as red connectors. The signaling network comprises the Wingless-related integration site (Wnt), Bone Morphogenetic Protein (BMP), Phosphotase and tensin homolog/phosphoinositide 3-kinase (PTEN/PI3K), Sonic Hedgehog Homolog (Hh), Tumor Necrosis Factor (TNF)-α, Interferon (IFN)-γ, RIP Kinase, nuclear factor kappa-light-chain-enhancer of activated B cells (NF-κB), Janus Kinase (JAK), Signal transducer and activator of transcription 3 (Stat3), and reactive oxygen species (ROSs), and Interleukin (IL) 6,10,13, and 15 signaling pathways. Full details of the implementation of these signaling relationships are presented in the Materials and Methods. (TIF) [file pcbi.1003507.s001.tif]

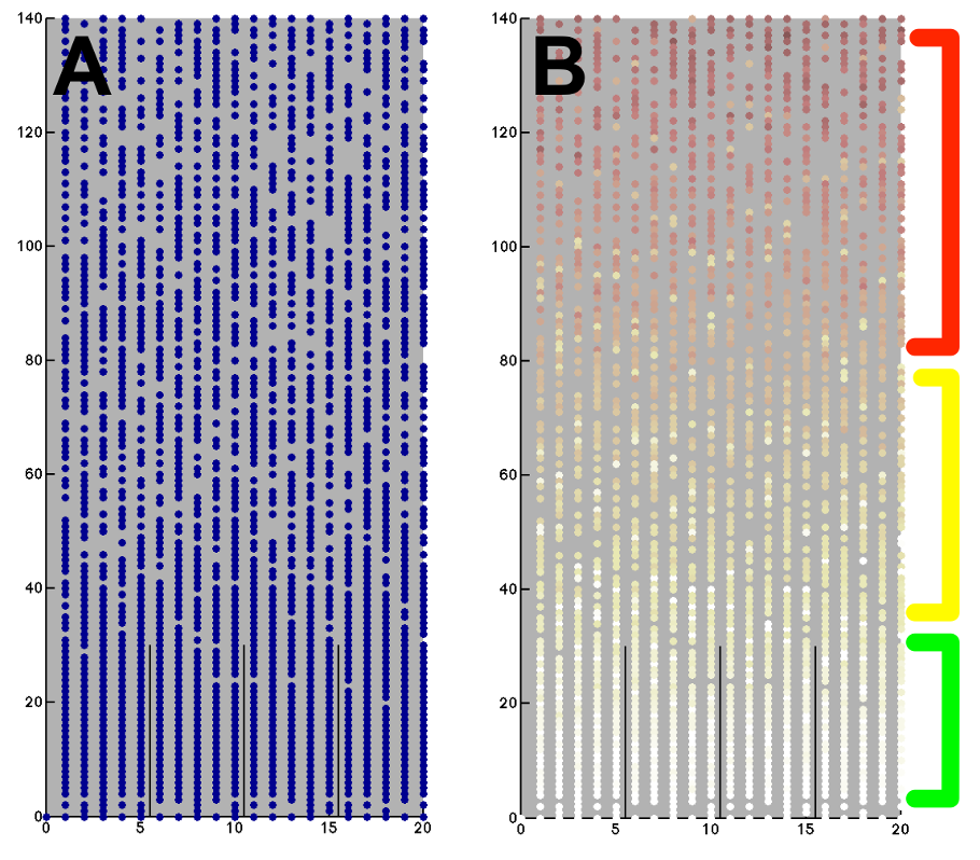

Supplement: Figure S2 — Simulation distributions of free Bone Morphogenetic Protein (BMP) and BMP binding to epithelial cells. Panel A shows the distribution of free molecular Bone Morphogenetic Protein (BMP) concentrations in SEGMEnT: free BMP is shown as blue, and having a uniform density throughout the tissue. This corresponds to histological data showing a uniform density of free BMP from the bottom of the crypt to the top of the villus (see Figure 1d from Ref [38] for a corresponding even distribution of blue-stained free BMP). Panel B displays the BMP activity gradient, representing binding of BMP with its receptor, generated by SEGMEnT, color coded as brown to match the histological staining in Figure 1f from Ref [38]. Zones of BMP activity closely match the pattern seen in published experiments (see Figure1f from Ref [38]) with simulated BMP activity/binding gradient matching experimental data with minimal BMP activity in the crypts (green brackets), a transition zone with BMP activity beginning at the crypt-villus junction (yellow brackets) and increasing until it maximizes at the tip of the villus (red brackets). (TIF) [file pcbi.1003507.s002.tif]
