# Supplementary material for: Investigation of Inflammation and Tissue Patterning in the Gut Using a Spatially Explicit General-Purpose Model of Enteric Tissue (SEGMEnT)
Source: PLoS Comput Biol. 2014 Mar 27;10(3):e1003507. doi: 10.1371/journal.pcbi.1003507 (PMC3967920; doi:10.1371/journal.pcbi.1003507)
Supplement: Table S1 — Primary molecular components included in SEGMEnT. (PDF) [file pcbi.1003507.s003.pdf]

Table S1: Primary Molecular Components included in SEGMEnT

Signal	Primary Function	Ref.
Wnt-β-catenin	Deactivates β-catenin destruction complex; maintain cells in undifferentiated state; direct migratory paths (via EphB receptor - Ephrin B ligand signaling); increase cellular proliferation.	[32-35][77]
Dkk1	Inhibits Wnt signaling by binding to and neutralizing Wnt receptors; balance Wnt self-stimulation to limit the size of the proliferative compartment of the crypt.	[79, 80]
Noggin	Binds to and neutralizes BMP molecules; protects the proliferative compartment from BMP signaling.	[32, 81]
BMP	Inhibits nuclear accumulation of β-catenin; up-regulated differentiation; limits crypt, villus growth.	[32, 38, 81, 82]
Hedgehog	Controls villus height through regulation of apoptosis; controls strength of GEC adhesion to villus; loss of Akt initiates signaling cascade leading to anoikis.	[43, 44, 83]
TGF-β-SMAD3	Increases apoptosis through up-regulation of SMAD3, an Akt inhibitor.	[84-86]
Akt	Controls villus height through regulation of apoptosis; controls strength of GEC adhesion to villus; loss of Akt initiates signaling cascade leading to anoikis.	[32, 79, 87-90]
PTEN/PI3K	Interrupts PI3K activity by converting PIP3 to PIP2; up-regulated by inflammation, down-regulates Hh (putative).	[32, 79, 87, 88]
NFκB	Anti-inflammatory response which inhibits apoptosis and necrosis. NFκB displays self-regulation through IκB.	[58][57, 91, 92]
TNF-α	Induces apoptosis and necrosis (if combined with RIP1); secreted primarily by inflammatory cells; up-regulates NFκB to prevent excess inflammation; inhibits proliferation through up-regulation of Dkk1.	[58-60, 93]
RIP	Induces cell death, but also up-regulates the expression of NFκB; modulates TNF functionality from apoptosis to necrosis.	[94-97][51]

JAK/STAT3	Various IL's up-regulate JAK, which activates STAT3. STAT3 leads to apoptosis in all cell types represented in SEGMEnT.	[61, 62]
IFN-γ	Prevents apoptosis through up-regulation of PI3K activity.	[98]
ROS	Induces cell death through necrosis; primarily secreted by neutrophils to assist in phagocytosis of necrotic debris.	[57, 91]
