# Supplementary material for: Investigation of Inflammation and Tissue Patterning in the Gut Using a Spatially Explicit General-Purpose Model of Enteric Tissue (SEGMEnT)
Source: PLoS Comput Biol. 2014 Mar 27;10(3):e1003507. doi: 10.1371/journal.pcbi.1003507 (PMC3967920; doi:10.1371/journal.pcbi.1003507)
Supplement: Table S2 — Rules for molecular interactions in SEGMEnT. (PDF) [file pcbi.1003507.s004.pdf]

Table S2: Rules for Molecular Interactions in SEGMEnT

Cytokine	Effect
1 u Wnt activity	Produces 1 u Dkk1
	Allows 1 u β-catenin to accumulate in the nucleus
	Produces 0.2 u Noggin
	Produces 0.2 u Wnt receptors, Wnt ligand
1 u Dkk1	Binds to and neutralizes 1 u Wnt receptors
1 u Noggin	Binds to and neutralizes 1 u BMP molecule
1 u SFRP	Binds to and neutralizes 1 u Wnt ligand
1 u β-catenin	Inhibits production of 0.1 u BMP receptors
	Produces 1 u EphB receptors
	Inhibits the production of 1 u Ephrin B ligand
1 u BMP activity	Prevents 1 u β-catenin from accumulating in nucleus
	Produces 1 u Hh
	Produces 1 u PTEN
1 u PTEN	Inhibits 1 u PI3K activity
	Inhibits Hh production of SMAD
1 u PI3K	Activates 1 u Akt
1 u Hh	Produces 1 u SFRP
	Produces 1 u TGF-β
1 u TGF-β	Produces 1 u SMAD
1 u SMAD	Causes immediate release of 0.2 u TGF-β
	Deactivates 1 u Akt
1 u ROS	Produces 1 u NFκB
1 u NFκB	Produces 1 u IκB
	Produces 1 u IFN-γ
	Produces 1 u TNF-α
	Prevents deactivation of 1 u Akt

1 u IκB	Prevents production of 0.75 u NFκB
1 u DAMP	Initiates production of 3 u NFκB
1 u IL10	Produces 1 u JAK
	Inhibits production of 0.33 u TNF-α
1 u IL15	Produces 1 u JAK
	Produces 1 u IFN-γ
1 u IL13	Produces 1 u JAK
1 u IL6	Produces 1 u JAK
1 u TNF-α	Produces 1 u RIP
	Produces 1 u Dkk1
	Produces 1 u PI3K activity
	Produces 1 u IL10
	Deactivates 1 u Akt
1 u RIP	Inhibits production of 1 u NFκB
1 u JAK	Initiates production of 1 u STAT3
1 u IFN-γ	Protects 1 u Akt from deactivation
1 u STAT3	Produces 2 u PTEN
	Deactivates 1 u Akt
