# Supplementary material for: Investigation of Inflammation and Tissue Patterning in the Gut Using a Spatially Explicit General-Purpose Model of Enteric Tissue (SEGMEnT)
Source: PLoS Comput Biol. 2014 Mar 27;10(3):e1003507. doi: 10.1371/journal.pcbi.1003507 (PMC3967920; doi:10.1371/journal.pcbi.1003507)
Supplement: Table S3 — Physical characteristics, published and derived time delays, and model-implemented time delays for signaling events involving molecular components included in SEGMEnT. (PDF) [file pcbi.1003507.s005.pdf]

Table S3: Physical characteristics, published and derived time delays, and model-implemented time delays for signaling events involving molecular components included in SEGMEnT

Protein	AA length	Num. Introns	Time Delay (min)	Time Delay (max)	Model Delay
Wnt	365	3	322	517	400
Dkk1	266	3	321	516	450
Nog	232	0	307	487	400
SHh	462	2	18	28	30
Ihh	411	2	17	27	30
TGF-β	390	6	37	67	45
Smad3	425	6	38	68	45
TNF-α	233	2	16	26	30
JAK	1154	24	128	248	150
RIP	671	9	52	97	60
NF-κB	415	8	46	86	60
IFN-γ	166	3	20	35	30
Stat3	770	23	122	237	150
JNK	427	10	56	106	75
IL6	212	4	26	46	30
IL10	178	4	25	45	30
IL13	146	3	20	35	30
IL15	162	9	50	95	60
