# Supplementary material for: Investigation of Inflammation and Tissue Patterning in the Gut Using a Spatially Explicit General-Purpose Model of Enteric Tissue (SEGMEnT)
Source: PLoS Comput Biol. 2014 Mar 27;10(3):e1003507. doi: 10.1371/journal.pcbi.1003507 (PMC3967920; doi:10.1371/journal.pcbi.1003507)
Supplement: Table S4 — Additional features and capabilities to be added to SEGMEnT. (PDF) [file pcbi.1003507.s006.pdf]

Table S4: Additional Features and Capabilities to be added to SEGMEnT

Feature	Function
Goblet Cells	More accurately reflect histology of mucosa; simulate maintenance of mucus layer; host-microbe interactions
Metabolism	Host-microbe dynamics involved in obesity [59-61], Type II Diabetes; simulations of disease states such as Environmental Enteropathy
Microbiome	Important to health, disease [64,65]; simulate host-microbe interactions; interplay between inflammation and microbiome
Enhanced Immunity	Implement advanced immunity controls to more accurately depict chronic inflammation
High Performance Computing (HPC)	Parallelize the model in order to represent larger, clinically realistic sections of tissue
