# Supplementary material for: Investigation of Inflammation and Tissue Patterning in the Gut Using a Spatially Explicit General-Purpose Model of Enteric Tissue (SEGMEnT)
Source: PLoS Comput Biol. 2014 Mar 27;10(3):e1003507. doi: 10.1371/journal.pcbi.1003507 (PMC3967920; doi:10.1371/journal.pcbi.1003507)
Supplement: Text S1 — Detailed description of underlying biology and its implementation in SEGMEnT. These materials provide a detailed description of the biological processes, modeling assumptions and implementation of the biological knowledge into SEGMEnT. These materials also provide descriptions of how the simulation experiments were performed. (PDF) [file pcbi.1003507.s007.pdf]

Supplementary Materials Text S1: Detailed Description of underlying biology and its implementation in SEGMEnT.

These materials provide a detailed description of the biological processes, modeling assumptions and implementation of the biological knowledge into SEGMEnT. These materials also provide descriptions of how the simulation experiments were performed.

Overall Architecture: Cell types and Tissue Topology

Cells

Gut epithelial cells (GECs) are the primary agent in SEGMEnT as they are the minimally sufficient actors needed to represent all of the necessary interactions required to describe intestinal homeostasis and inflammatory processes. We choose GECs as the agents because cells are represented as discrete units of fixed size that can move, divide, differentiate, and communicate with each other and their environment through signaling events. A typical crypt in healthy ileal tissue will contain 500 epithelial cells; approximately 2000 cells occupy a typical villus.

Cells interact with each other solely through signaling, however they can impede each other's motion; two cells are not allowed to occupy the same space, though they do not exert a true mechanical force on each other. When reacting to inflammation and certain other signals, GECs are considered to have an abundance of receptors, and thus react proportionately to any amount of cytokine.

Signaling Network

Fig. 3 depicts the relationship between the process, topology, and output of SEGMEnT. All of the cellular "decisions" occur in the crypt, shortly after stem cell proliferation. A

fate is quickly chosen, and as the cell exits the proliferative zone of high Wingless-related integration site (Wnt) activity, it differentiates. The cell then continues to climb the villus (directed by the EphB receptor-Ephrin B ligand gradient) until it de-attaches from the base membrane and undergoes anoikis, a special type of apoptosis [89, 90].

Fig. S1 depicts the signaling pathway that controls GEC behavior in SEGMEnT. Wnt signaling self-stimulates in GECs and up-regulates Noggin. Wnt activity also prevents the destruction of β-catenin, which is responsible for defining a cell's migratory path through the EphB/Ephrin-B signaling pathway. Noggin inhibits bone morphogenic protein (BMP) activity by binding to and neutralizing BMP molecules [32, 81]. BMP activity opposes Wnt activity through up-regulating Sonic Hedgehog Homolog (Hh) signaling. BMP activity also up-regulates Phosphotase and tensin homolog (PTEN), which indirectly causes apoptosis. The signaling molecules and their associated functions are listed in Table 1.

Agent Details: There are five cell types represented in the model: intestinal stem cells (ISCs), undifferentiated transit amplifying (TA) cells, differentiated enterocytes, neutrophils, and macrophages.

Intestinal Stem Cells (ISC): Each crypt contains 4 ISCs in an azimuthally symmetric distribution located 4 cell lengths above the base of the crypt [32]. In order to maintain homeostasis, ISC's divide asymmetrically approximately every 2 hours. If an ISC undergoes necrosis, it typically leads to the total death of the crypt as SEGMEnT lacks a mechanism for ISC renewal.

Transit Amplifying (TA) Cells: Undifferentiated cells follow a decreasing EphB receptor gradient as they migrate up the crypt with a speed of approximately 15 μm/hr. TA cells divide probabilistically with $P_{(divide)} = 0.15$/hr, thus, a TA cell created by an ISC will divide an average of approximately four times as it traverses 25 cell lengths (1 cell length = 10 μm). This corresponds to a crypt that has a depth of 300 μm, typical in the human ileum. TA cells are twice as susceptible to necrosis as differentiated enterocytes.

Enterocytes: Differentiated enterocytes follow an increasing Ephrin-B ligand gradient as they migrate up the villus. Enterocytes' sole function is to interact with the signaling network. Enterocytes respond to inflammatory signals with 0.25 the strength of an inflammatory cell.

Neutrophils, Macrophages: Upon exposure to damage-associated molecular pattern molecules (DAMPs), blood vessels start secreting neutrophils and macrophages. Every 15 minutes, a neutrophil or macrophage has the opportunity to adhere to the tissue outside of the blood vessel with a probability based on the amount of DAMP present: $P(adhere) = c_a[DAMP]$ where $c_a = (10\,u)^{-1}$. Neutrophils have a limited signaling network and only secrete reactive oxygen species (ROS) in response to pro-inflammatory stimuli, while macrophages utilize the full inflammatory signaling network.

Neutrophils and macrophages clear necrotic debris through phagocytosis; a single neutrophil or macrophage can clear one cell's worth of necrotic debris in approximately

10 hours, though in plural, they typically clear a dead cell in less than one hour. Neutrophils and macrophages have finite lifetimes of 2 days, though can undergo apoptosis earlier than this if they aggregate sufficient Signal transducer and activator of transcription 3 (STAT3).

Implementation of Signaling Networks

Absolute measurements of signaling molecules are not required in SEGMEnT; relative chemokine concentrations dictate the system's behavior, therefore, all signaling molecules are measured in arbitrary units.

Morphogenesis Pathways (see Blue Components in Figure S1)

Wnt-β-catenin: The Wnt-β-catenin signaling pathway is one of the most fundamental signaling pathways in the gut [32]. As the functionality of non-canonical Wnt signaling is still a topic of open research, we restrict our modeling of Wnt signaling to the canonical Wnt signaling; that is, Wnt ligands 3,6, and 9B complex with their associated receptors: Frizzled 5,6, 7 and LRP5 and 6. Wnt signaling is responsible for maintaining cells in an undifferentiated state, directing migratory paths, increasing cellular proliferation, and influencing cell lineage decisions [32-35].

The origin of the Wnt source is unknown, though there is evidence that Wnt proteins originate in the mesenchyme directly adjacent to the base of the crypt [35]. It is also possible that undifferentiated cells can produce Wnt proteins as part of a self-stimulation process. In order to most accurately represent the current knowledge about

Wnt signaling, SEGMEnT contains a single Wnt source at the base of the crypt. This point source forms an exponentially decaying gradient of Wnt activity. We allow this Wnt source to self-stimulate linearly based on the number of putative Wnt producing cells. A further self-stimulation mechanism is introduced in which cells in Wnt-active tissue regions secrete Wnt proteins to their surrounding cells as well as form new Wnt receptors.

Recent studies have suggested that Wnt signaling also drives a time-delayed Wnt suppressor [79, 80]. Dickkopf-related protein 1 (Dkk1), a downstream target of β-catenin signaling, down-regulates Wnt activity by binding to and neutralizing Wnt receptors. This mechanism prevents infinite crypt growth with a self-stimulating Wnt source.

A cell's immediate response to Wnt signaling is the inactivation of its β-catenin destruction complex. This allows β-catenin to accumulate in the nucleus, bind to the lymphoid enhancer factor (LEF) or T cell factor (TCF) transcription factors, which leads to the activation of target genes. We use intra-cellular β-catenin as the marker for differentiation; when a cell exits an area with active Wnt signaling, the β-catenin destruction complex activates and begins to destroy that cell's β-catenin. When all of the β-catenin has been destroyed, the cell undergoes a differentiation process in which it becomes either an absorptive enterocyte or secretory goblet cell [32].

Wnt signaling directs the cellular migratory paths through the construction of an EphB receptor/Ephrin-B ligand gradient [99, 100]. β-catenin up-regulates the formation of EphB receptors and down-regulates the expression of Ephrin-B ligands. It is well known that EphB receptors and Ephrin-B ligands display a repulsive signaling interaction. This repulsive interaction prevents differentiated cells from re-entering the

crypt and establishes a migratory path from the base of the crypt to the tip of the villus. It should be noted that EphB expression extends beyond the region where cells have accumulated β-catenin; this is possibly due to the continuing translation of EphB mRNAs after the transcription of their associated genes or structural instabilities generated by continued exposure to Ephrin-B ligand [100].

Wnt signaling also up-regulates the expression of Noggin, a BMP inhibitor [32, 81]. Noggin functions by binding to and neutralizing BMP molecules. SEGMEnT models this with a Noggin point source at the base of the crypt. Noggin is up-regulated by Wnt, however epithelial cells do not secrete Noggin, so this self-stimulation in limited to the source at the base of the crypt.

A cell begins with 30 u of Wnt receptors. If a cell is engaged in active Wnt signaling, the cell produced Wnt receptors at a rate of 2 receptors/hr. When 1 u of Wnt ligand complexes with 1 u of Wnt receptors, the cell begins the transcription for 1 u of Dkk1, 0.2 units of Noggin, and 0.5 u of Wnt ligand. Active Wnt signaling prevents the otherwise automatic destruction of β-catenin. Active Wnt signaling also allows for the maintenance of EphB receptors – as long as the β-catenin destruction complex is inactivated, EphB receptors are created at a rate of 2 u/hr. 1 u of Dkk1 inhibits Wnt activity by binding to and neutralizing 1 u of Wnt receptors. 1 u of secreted frizzled-related protein 1 (SFRP1) inhibits Wnt activity by binding to and neutralizing 1 u of Wnt molecule.

<u>BMP:</u> BMP signaling serves to act as a balance against Wnt signaling [32]. BMPs are members of the TGF-β family and function by binding to the complex of BMP receptor

type 1 and BMP receptor type 2 [81]. BMP signaling inhibits the ability of beta-catenin to accumulate in the nucleus [38, 82]. BMP signaling up-regulates differentiation and limits crypt growth by increasing the rate of β-catenin destruction via the up-regulation of Hh [45], and indirectly controls cell lifetimes and apoptosis through up-regulation of PTEN [32]. BMP molecules exist in a roughly even distribution throughout the epithelial tissue [38], though the receptors form a gradient beginning at the crypt-villus junction and maximizing at the tip of the villus. In order to model this, we consider BMP receptors to be down-regulated by β-catenin in a similar manner to Ephrin-B ligand.

BMP molecule is continuously secreted into the environment such that the un-inhibited concentration of BMP is approximately 200 u/μm^2. BMP receptors are created at a rate of 2 u/hr when the β-catenin destruction complex is inactivated. When 1 u of BMP molecule is complexed with 1 u of BMP receptor, the cell begins the transcription process for 1 u of Hh and 1 u of PTEN. 1 u of Noggin binds to and neutralizes 1 u of BMP molecule.

Hh: Hedgehog signaling separates differentiated cells from the proliferative crypt and serves to fine-tune the location of the crypt-villus junction [83]. Differentiated cells secrete Hh molecules into the mesenchyme. Hh molecules bind to Patched 1 and 2 receptors. This induces expression of SFRP1, a molecule, which inhibits Wnt by binding to and neutralizing Wnt proteins [43, 44]. In SEGMEnT, we model this by allowing SFRP1 to diffuse downward from the villus to the base of the crypt (if a sufficient amount has been produced). This forms an exponentially decaying gradient, and counteracts both the primary Wnt source as well as the secondary Wnt self-stimulation.

Hh signaling also provides a redundant apoptotic regulatory mechanism to assist the PTEN pathway. Hh activity induces transforming growth factor beta (TGF-β) activity [58], which leads to the production of SMAD3, a known apoptotic agent [84-86].

Hh receptors are considered to exist in abundance, and thus a cell can receive an arbitrary amount of Hh signal. When 1 u of Hh molecule complexes with its associated receptors, the cell begins the transcription for 1 u of SFRP1, and causes the immediate release of 1 u of TGF-β, which leads to the transcription of 1 u of SMAD3.

Inflammatory Pathways (see Orange Components in Figure S1)

PTEN/PI3K/Akt: The PTEN/PI3K pathway is primarily responsible for regulating apoptosis and villus height [32, 87, 88]. Phosphatidylinositide 3-kinase (PI3K) is directly responsible for the activation of Akt, the molecule responsible for cellular adhesion to the underlying matrix. PI3K phosphorylates Phosphatidylinositol 4,5-bisphosphate (PIP2) into Phosphatidylinositol (3,4,5)-triphosphate (PIP3), leading to the activation of Akt. PTEN inhibits PI3K/Akt activity by converting PIP3 to PIP2, thus interrupting the Akt activation process [79]. PTEN is up-regulated by BMP activity, thus when BMP activity reaches some critical value, PI3K activity is sufficiently dampened that the cell can de-attach from the villus and die. It has been demonstrated that cells become more loosely bound as they migrate up the villus; when they detach, the cells undergo anoikis, a special type of apoptosis [89, 90]. By regulating apoptosis, the PTEN/PI3K pathway also controls villus height. We posit that PTEN is up-regulated by inflammation (for which we use STAT3 as a proxy), and it is this up-regulation that is responsible for the known down-regulation of Hh [101].

Each cell is considered to start with 250 units of Akt and PI3K. Each unit of PI3K corresponds to an active unit of Akt. Absent external signaling, a cell will maintain its level of PI3K. 1 u of PTEN eliminates 1 u of PI3K, leading to the inactivation of 1 u of Akt. 1 u of Akt is inactivated by 1 u of Mothers against decapentaplegic homolog 3 (SMAD3), 1 u of tumor necrosis factor alpha (TNF-α), or 1 u of Stat3. This inactivation can be prevented by either Interferon gamma (IFN-γ) or nuclear factor kappa-light-chain-enhancer of activated B cells (NF-κB) where 1 u of either will protect 1 u of Akt. If all of a cell's Akt is inactivated, the cell undergoes apoptosis and is removed from the simulation.

TLR4**:** In SEGMEnT, toll-like receptor 4 (TLR4) signaling represents the beginning of the inflammatory cascade. TLR4 signaling increases cellular survivability by directly up-regulating PI3K and NF-κB; TLR4 signaling also guides cell's to death by necrosis or apoptosis through the direct up-regulation of RIP and the indirect up-regulation of TNF-α and the recruitment of inflammatory cells, which secrete various interleukins causing a buildup of STAT3 [57]. TLR4 receptors are activated by DAMP. 1 u of activated TLR4 receptors causes the production of 1 u of receptor interacting protein (RIP) and 1 u of NF-κB.

TNF-α: TNF-α plays a central role in the inflammatory module to SEGMEnT. TNF-α is produced by all cell types represented in the model, though primarily by the inflammatory neutrophils and macrophages [58]. TNF-α serves to: induce either apoptosis or necrosis, depending on the corresponding amount of RIP1 [58, 59]; inhibit

cellular proliferation through the up-regulation of Dkk1 [60]; up-regulate itself through the indirect activation of NF-κB [58]; and down-regulate itself through the induction of interleukin-10 (IL-10) [93]. TNF-α signaling is mediated through its associated receptors, tumor necrosis factor receptor 1 and 2 (TNFR1 and TNFR2). SEGMEnT considers both of these receptors to exist in abundance and thus a cell will respond to any amount of TNF that exists within the reach of the cell's receptors. Self-inhibitory mechanisms within the inflammatory signaling network prevent any runaway TNF-signaling from sustained inflammation. 1 u of TNF-α causes the production of 0.75 u of IL10, 1 u of IL13, 1 u of RIP, 1 u of NF-κB, and 1 u of Dkk1. 1 u of IL10 will inhibit the production of 1 u of TNF-α.

<u>IFN-γ:</u> IFN-γ plays seemingly contradictory roles in the model. IFN-γ inhibits cellular proliferation via the induction of Dkk1, but also inhibits apoptosis by enhancing the activity of PI3K [98].

<u>NF-κB/IκB:</u> NF-κB also plays a central role in SEGMEnT's inflammatory module. NF-κB's primary function is to inhibit apoptosis [92]. NF-κB activity can be induced in SEGMEnT by TNF signaling, TLR4 signaling, or contact with reactive oxygen species (ROSs) secreted by inflammatory cells [57, 91]. The up-regulation of TNF serves to create a self-propagating inflammatory loop, which is balanced out by the down-regulation of NF-κB by RIP and nuclear factor of kappa light polypeptide gene enhancer in B-cells inhibitor (IκB). IκB production is induced by NF-κB activity and prevents runaway inflammation due to forward-feedback interactions with other elements of the

inflammatory network. 1 u of NF-κB leads to the production of 1 u of TNF-α, 1 u of IFN-γ, 1 u of IL-6, and 0.75 u of IκB. 1 u of IκB prevents the transcription of 1 u of NF-κB.

JAK/STAT3: STAT3 is phosphorylated and activated by Janus kinase (Jak). STAT3 induces the expression of several regulatory subunits of PI3K, which then reduces the overall level of Akt activity, and in doing so, increases the likelihood that the cell will undergo apoptosis [61, 62]. 1 u of JAK is created in response to 1 u of IL-6, IL-10, IL-13, or IL-15. 1 u of JAK leads to the production of 1 u of Stat3. 1 u of Stat3 leads to the production of 4 u of PTEN.

RIP Kinase: RIP Kinase is another molecule that plays contradictory roles in the model. RIP1 induces cell death through necrosis, but at the same time up-regulates NF-κB expression, increasing a cell's survivability [94, 95]. RIP is secreted in response to TNF exposure or TLR4 signaling [94]. RIP binds to TNF receptors and in doing so change the form of cell death from apoptosis to necrosis [96]. Biologically, the polyubiquitination of RIP is induced upon RIP binding to the TNF receptor, TNFR1. This causes the activation of NF-κB. When this ubiquitination is reversed, the functionality of RIP is changed to promote cell death through necrosis [95, 97]. 1 u of RIP prevents the transcription of 1 u of NF-κB. 1 u of RIP has a necrotic contribution of 1 (when combined with an equal amount of TNF-α. 1 u of ROS has a necrotic contribution of 10. When an enterocyte has a necrotic value of 100, it undergoes necrosis.

Interleukins: IL-6, IL-10, IL-13, and IL-15 are represented in the model. This class of molecules is secreted primarily by inflammatory cells in response to TLR4 signaling. IL-10 acts as an anti-inflammatory agent by inhibiting the activity of TNF-α. IL-10 signaling is regulated by TNF as well as TLR4 signaling [62]. IL-6 disposes a cell towards apoptosis by up-regulating the expression of STAT3 [98, 102]. IL-13 is also stimulated by TNF signaling and encourages apoptosis [103]. IL-15 prevents apoptosis through an up-regulation of IFN-γ [104]. IL-6, IL-10, IL-13, and IL-15 are secreted at a constant rate (unless modified by external signaling) of 2u/hr by inflammatory signals in response to TLR4 signaling.

Signaling Time Delays: The delay from signaling event to its effect is a result of transcriptional, translational, and transportation delays [105, 106]. Experiment is combined with accepted measurements regarding these delays to estimate signaling time delays. mRNA is transcribed at a rate of 20 nucleotides/sec; there is an approximately 5 minute delay as the RNA is transferred from the nucleus to the cytoplasm; the translation rate when synthesizing the target protein is 6 nucleotides/sec; each intron requires 5-10 minutes to remove. As these well-known delays are not sufficient to reproduce experiment, an experimental delay from the Wnt signaling event to β-catenin related transcription of 5-8 hours is added. Delays used in SEGMEnT are shown in Table 3.

Diffusion of Secreted Mediators

SEGMEnT implements a 1st order discrete approximation to the diffusion equation:

$$\frac{\partial \phi(\vec{x},t)}{\partial t} = D\nabla^2 \phi(\vec{x},t).$$

As we are on a 2d grid, this becomes:

$$\frac{\partial\phi(\vec{x},t)}{\partial t} \approx D\left[\phi_{i+1,j}(t) + \phi_{i-1,j}(t) + \phi_{i,j+1}(t) + \phi_{i,j-1}(t) - 4\phi_{i,j}(t)\right],$$

where D is the diffusion constant. We have adopted the notation $\phi_{i+1,j}(t) = \phi(x + \Delta x, y, t)$. This leads to the final approximation:

$$\phi_{i,j}(t + \Delta t) = \phi_{i,j}(t) + D\Delta t[\phi_{i+1,j}(t) + \phi_{i-1,j}(t) + \phi_{i,j+1}(t) + \phi_{i,j-1}(t) - 4\phi_{i,j}(t)].$$

We approximate the diffusion constant for all molecules to be 100 $\mu m^2 s^{-1}$, equal to that of BMP-2 molecule [107].

Simulation Details

A 0.64 mm^2 section of ileal tissue comprising 8 crypts and 8 villi was simulated for all simulation experiments.

Wnt Inhibitor

SEGMEnT simulates a Wnt inhibitor by decreasing the amount of Wnt produced by the Wnt source at the base of the crypt by 25% per hour, decreasing the amount of Noggin produced at the Wnt source by 25% per hour, and eliminating the ability of intestinal cells to have self-stimulating Wnt activity.

Hh Inhibitor

SEGMEnT simulates an Hh inhibitor by removing the ability to synthesize Hh from the differentiated enterocytes. This allows the crypt to grow by eliminating the Wnt inhibitor, SFRP1. Villus height is maintained by the redundant PTEN apoptosis pathway.

PTEN Inhibitor

SEGMEnT simulates a PTEN inhibitor by removing the ability to synthesize PTEN from the differentiated enterocytes. This forces the Hh pathway to become the sole regulator of villus height.

Local Mucosal Wound

To simulate tissue injury/trauma SEGMEnT applies 10 u of DAMP and 10 u of ROS to the villi in the simulation. This causes the immediate necrosis of all affected cells. Blood vessels register the inflammation, causing macrophages and neutrophils to activate and begin to clear the necrotic tissue.

Intestinal Ischemia/Reperfusion

Ischemia is represented by deactivating all blood vessels in the affected tissue; this results in a 70% decrease in ISC proliferation, a 90% decrease in TA cell proliferation, a 10% increase in inflammatory potential per hour in GEC's. During the ischemic period, TA cells and differentiated enterocytes increase their inflammatory response [108] such that their response has 0.8 the strength of an inflammatory cell. Crypts rapidly shrink after the onset of ischemia, due primarily to the retarded proliferation [108-110]. Ischemic GECs produce and secrete fatty acid binding protein (FABP). This compound accumulates during the ischemic period, and upon reperfusion acts to initiate sloughing of associated GECs [55]. Upon reperfusion ischemic cells produce a series of pro-inflammatory factors (platelet activating factor (PAF), interleukin-8 (IL-8) and various endothelial adhesion

factors) currently aggregated as “neutrophil aggregation factor,” that attracts and activates neutrophils and initiates the inflammation seen with reperfusion. In the absence of sloughing the ischemic/necrotic GECs remain on the villus for a longer length of time, continuing to secrete damage associated molecular pattern molecules (DAMPs) and neutrophil aggregation factors in response to the reperfusion inflammation until they have been removed via phagocytosis. Inflammatory cytokines thus generated build up in the crypt until the undifferentiated cells begin to necrose. After a critical mass of cells has necrosed, the inflammation becomes self-sustaining (i.e., “cytokine storm”). Upon reperfusion, proliferation rates return to normal and the system enters a period of crypt hyperplasia as there are fewer differentiated cells to antagonize the Wnt differentiation pathway through Hh. As the villus regrows, the crypt returns to a normal size and the system returns to homeostasis.

Colonic Metaplasia of the Ileum

Simulation of the pathogenesis of colonic metaplasia in SEGMEnT is based on the hypothesis that there is a chronic low-level inflammatory stimulus to the enteric mucosa [27], and implemented as the presence of DAMP as a stimulus to GEC TLR4 receptors. 10 u of DAMP are applied to each cell on the villus, and this is maintained indefinitely.

Additional References for Supplementary Text S1:

79. Grossmann J, Walther K, Artinger M, Kiessling S, Scholmerich J (2001) Apoptotic signaling during initiation of detachment-induced apoptosis ("anoikis") of primary human intestinal epithelial cells. Cell Growth Differ 12: 147-155.
80. Frisch SM, Screaton RA (2001) Anoikis mechanisms. Curr Opin Cell Biol 13: 555 562.
81. von Bubnoff A, Cho KW (2001) Intracellular BMP signaling regulation in vertebrates: pathway or network? Dev Biol 239: 1-14.

82. Colla S, Zhan F, Xiong W, Wu X, Xu H, et al. (2007) The oxidative stress response regulates DKK1 expression through the JNK signaling cascade in multiple myeloma plasma cells. Blood 109: 4470-4477.
83. Niida A, Hiroko T, Kasai M, Furukawa Y, Nakamura Y, et al. (2004) DKK1, a negative regulator of Wnt signaling, is a target of the beta-catenin/TCF pathway. Oncogene 23: 8520-8526.
84. Batlle E, Henderson JT, Beghtel H, van den Born MM, Sancho E, et al. (2002) Beta catenin and TCF mediate cell positioning in the intestinal epithelium by controlling the expression of EphB/ephrinB. Cell 111: 251-263.
85. Holmberg J, Genander M, Halford MM, Anneren C, Sondell M, et al. (2006) EphB receptors coordinate migration and proliferation in the intestinal stem cell niche. Cell 125: 1151-1163.
86. Auclair BA, Benoit YD, Rivard N, Mishina Y, Perreault N (2007) Bone morphogenetic protein signaling is essential for terminal differentiation of the intestinal secretory cell lineage. Gastroenterology 133: 887-896.
87. van den Brink GR (2007) Hedgehog signaling in development and homeostasis of the gastrointestinal tract. Physiol Rev 87: 1343-1375.
88. Cao Y, Liu X, Zhang W, Deng X, Zhang H, et al. (2009) TGF-beta repression of Id2 induces apoptosis in gut epithelial cells. Oncogene 28: 1089-1098.
89. Undevia NS, Dorscheid DR, Marroquin BA, Gugliotta WL, Tse R, et al. (2004) Smad and p38-MAPK signaling mediates apoptotic effects of transforming growth factor-beta1 in human airway epithelial cells. Am J Physiol Lung Cell Mol Physiol 287: L515-524.
90. Derynck R, Zhang YE (2003) Smad-dependent and Smad-independent pathways in TGF-beta family signalling. Nature 425: 577-584.
91. Langlois MJ, Roy SA, Auclair BA, Jones C, Boudreau F, et al. (2009) Epithelial phosphatase and tensin homolog regulates intestinal architecture and secretory cell commitment and acts as a modifier gene in neoplasia. FASEB J 23: 1835 1844.
92. Lee G, Goretsky T, Managlia E, Dirisina R, Singh AP, et al. (2010) Phosphoinositide 3-kinase signaling mediates beta-catenin activation in intestinal epithelial stem and progenitor cells in colitis. Gastroenterology 139: 869-881, 881 e861-869.
93. Sherman AE, Zavros Y (2011) Role of Sonic Hedgehog signaling during progression from inflammation to cancer in the stomach. World J Gastrointest Pathophysiol 2: 103-108.
94. Stenvinkel P, Ketteler M, Johnson RJ, Lindholm B, Pecoits-Filho R, et al. (2005) IL-10, IL-6, and TNF-alpha: central factors in the altered cytokine network of uremia--the good, the bad, and the ugly. Kidney Int 67: 1216-1233.
95. Nava P, Koch S, Laukoetter MG, Lee WY, Kolegraff K, et al. (2010) Interferon gamma regulates intestinal epithelial homeostasis through converging beta-catenin signaling pathways. Immunity 32: 392-402.
96. Edelblum KL, Yan F, Yamaoka T, Polk DB (2006) Regulation of apoptosis during homeostasis and disease in the intestinal epithelium. Inflamm Bowel Dis 12: 413 424.
97. Ghosh S, May MJ, Kopp EB (1998) NF-kappa B and Rel proteins: evolutionarily conserved mediators of immune responses. Annu Rev Immunol 16: 225-260.

98. Meylan E, Tschopp J (2005) The RIP kinases: crucial integrators of cellular stress. Trends Biochem Sci 30: 151-159.
99. Declercq W, Vanden Berghe T, Vandenabeele P (2009) RIP kinases at the crossroads of cell death and survival. Cell 138: 229-232.
100. Galluzzi L, Kepp O, Kroemer G (2009) RIP kinases initiate programmed necrosis. J Mol Cell Biol 1: 8-10.
101. Galluzzi L, Vanden Berghe T, Vanlangenakker N, Buettner S, Eisenberg T, et al. (2011) Programmed necrosis from molecules to health and disease. Int Rev Cell Mol Biol 289: 1-35.
102. Jin X, Zimmers TA, Zhang Z, Pierce RH, Koniaris LG (2010) Interleukin-6 is an important in vivo inhibitor of intestinal epithelial cell death in mice. Gut 59: 186 196.
103. Heller F, Florian P, Bojarski C, Richter J, Christ M, et al. (2005) Interleukin-13 is the key effector Th2 cytokine in ulcerative colitis that affects epithelial tight junctions, apoptosis, and cell restitution. Gastroenterology 129: 550-564.
104. Inoue S, Unsinger J, Davis CG, Muenzer JT, Ferguson TA, et al. (2010) IL-15 prevents apoptosis, reverses innate and adaptive immune dysfunction, and improves survival in sepsis. J Immunol 184: 1401-1409.
105. Audibert A, Weil D, Dautry F (2002) In vivo kinetics of mRNA splicing and transport in mammalian cells. Mol Cell Biol 22: 6706-6718.
106. Neugebauer KM (2002) On the importance of being co-transcriptional. J Cell Sci 115: 3865-3871.
107. Lander AD, Nie Q, Wan FY (2002) Do morphogen gradients arise by diffusion? Dev Cell 2: 785-796.
108. Feinman R, Deitch EA, Watkins AC, Abungu B, Colorado I, et al. (2010) HIF-1 mediates pathogenic inflammatory responses to intestinal ischemia-reperfusion injury. Am J Physiol Gastrointest Liver Physiol 299: G833-843.
109. Shima Y, Tajiri T, Taguchi T, Suita S (2006) Increased expression of c-fos and c-jun in the rat small intestinal epithelium after ischemia-reperfusion injury: a possible correlation with the proliferation or apoptosis of intestinal epithelial cells. J Pediatr Surg 41: 830-836.
110. Hinnebusch BF, Ma Q, Henderson JW, Siddique A, Archer SY, et al. (2002) Enterocyte response to ischemia is dependent on differentiation state. J Gastrointest Surg 6: 403-409.
